# Single-cell CRISPR immune screens reveal immunological roles of tumor intrinsic factors

**DOI:** 10.1093/narcan/zcac038

**Published:** 2022-12-09

**Authors:** Jiakai Hou, Shaoheng Liang, Chunyu Xu, Yanjun Wei, Yunfei Wang, Yukun Tan, Nidhi Sahni, Daniel J McGrail, Chantale Bernatchez, Michael Davies, Yumei Li, Rui Chen, S Stephen Yi, Yiwen Chen, Cassian Yee, Ken Chen, Weiyi Peng

**Affiliations:** Department of Biology and Biochemistry, University of Houston, Houston, TX, USA; Department of Bioinformatics and Computational Biology, The University of Texas MD Anderson Cancer Center, Houston, TX, USA; Department of Computer Science, Rice University, Houston, TX, USA; Department of Biology and Biochemistry, University of Houston, Houston, TX, USA; Department of Bioinformatics and Computational Biology, The University of Texas MD Anderson Cancer Center, Houston, TX, USA; Department of Melanoma Medical Oncology, The University of Texas MD Anderson Cancer Center, Houston, TX, USA; Department of Bioinformatics and Computational Biology, The University of Texas MD Anderson Cancer Center, Houston, TX, USA; Department of Bioinformatics and Computational Biology, The University of Texas MD Anderson Cancer Center, Houston, TX, USA; Department of Epigenetics and Molecular Carcinogenesis, The University of Texas MD Anderson Cancer Center, Houston, TX, USA; Center for Immunotherapy and Precision Immuno-Oncology, Cleveland Clinic, Cleveland, OH, USA; Department of Melanoma Medical Oncology, The University of Texas MD Anderson Cancer Center, Houston, TX, USA; Department of Melanoma Medical Oncology, The University of Texas MD Anderson Cancer Center, Houston, TX, USA; Department of Molecular and Human Genetics, Baylor College of Medicine, Houston, TX, USA; Department of Molecular and Human Genetics, Baylor College of Medicine, Houston, TX, USA; Department of Oncology, Livestrong Cancer Institutes, and Department of Biomedical Engineering, The University of Texas at Austin, Austin, TX, USA; Interdisciplinary Life Sciences Graduate Programs (ILSGP) and Oden Institute for Computational Engineering and Sciences (ICES), The University of Texas at Austin, Austin, TX, USA; Department of Bioinformatics and Computational Biology, The University of Texas MD Anderson Cancer Center, Houston, TX, USA; Department of Melanoma Medical Oncology, The University of Texas MD Anderson Cancer Center, Houston, TX, USA; Department of Immunology, The University of Texas MD Anderson Cancer Center, Houston, TX, USA; Department of Bioinformatics and Computational Biology, The University of Texas MD Anderson Cancer Center, Houston, TX, USA; Department of Biology and Biochemistry, University of Houston, Houston, TX, USA

## Abstract

Genetic screens are widely exploited to develop novel therapeutic approaches for cancer treatment. With recent advances in single-cell technology, single-cell CRISPR screen (scCRISPR) platforms provide opportunities for target validation and mechanistic studies in a high-throughput manner. Here, we aim to establish scCRISPR platforms which are suitable for immune-related screens involving multiple cell types. We integrated two scCRISPR platforms, namely Perturb-seq and CROP-seq, with both *in vitro* and *in vivo* immune screens. By leveraging previously generated resources, we optimized experimental conditions and data analysis pipelines to achieve better consistency between results from high-throughput and individual validations. Furthermore, we evaluated the performance of scCRISPR immune screens in determining underlying mechanisms of tumor intrinsic immune regulation. Our results showed that scCRISPR platforms can simultaneously characterize gene expression profiles and perturbation effects present in individual cells in different immune screen conditions. Results from scCRISPR immune screens also predict transcriptional phenotype associated with clinical responses to cancer immunotherapy. More importantly, scCRISPR screen platforms reveal the interactive relationship between targeting tumor intrinsic factors and T cell-mediated antitumor immune response which cannot be easily assessed by bulk RNA-seq. Collectively, scCRISPR immune screens provide scalable and reliable platforms to elucidate molecular determinants of tumor immune resistance.

## Graphical Abstract

**Graphical Abstract:**
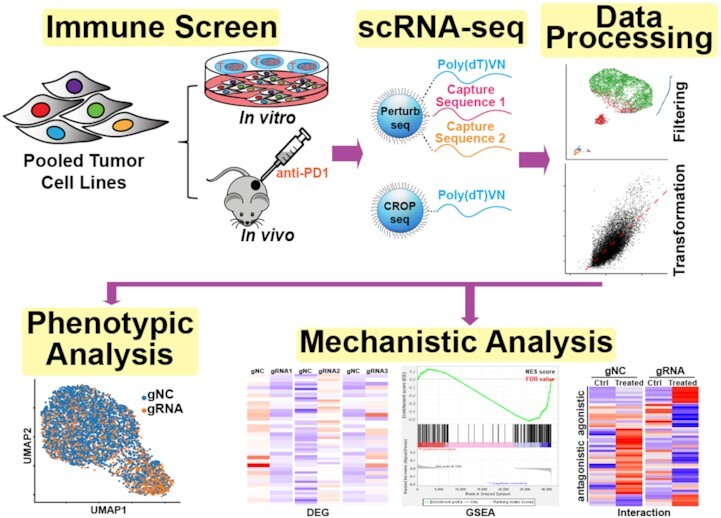
scCRISPR immune screens provide scalable and reliable platforms to elucidate tumor immune evasion mechanisms. A brief summary of experimental procedures and bioinformatics analysis pipelines for scCRISPR immune screens are illustrated.

## INTRODUCTION

RNA interference and CRISPR/Cas9 technologies have been widely utilized to interrogate the biology of genes in an unbiased, high throughput manner. Genetic screens developed using these technologies are greatly accelerating discoveries of key resistance mechanisms to cancer treatments, including cancer immunotherapy (1–3). A broad range of molecules that are involved in regulating tumor resistance to immune cell-mediated killing (4–7) and trafficking of immune cells to tumors (8,9) have been identified. Integration of genetic screen results with multi-omic datasets from cancer patients further guides the selection of immune-oncology (IO) combinations with clinical relevance (10). However, it is time- and resource-intensive to individually validate these immune factors *in vivo* and elucidate their working mechanisms. Thus, it is desirable to develop a high-throughput process to characterize these factors in parallel.

Recent efforts have been taken to pair CRISPR/Cas9 perturbation with powerful single-cell RNA sequencing (scRNA-seq) to systematically characterize transcriptional phenotypes caused by individual perturbations. These efforts led to the establishment of several independent single-cell CRISPR (scCRISPR) screen platforms, including Perturb-seq (11), CROP-seq, (12) and CRISP-seq (13). Although these three major scCRISPR screen platforms share many similarities, such as using the workflow of 3′ end scRNA-seq technology, they utilize distinct approaches to capture and assign gRNA reads to individual cells. Perturb-seq requires modification of gRNA scaffolds in gRNA-expressing vectors and single-cell gel beads. By incorporating capture sequences in gRNA scaffolds and their complementary primers into single-cell gel beads, both gRNAs and mRNAs from individual cells can be simultaneously captured (14,15). gRNAs, transcribed by RNA polymerase (RNAP) III, are directly sequenced and used to assign the gRNA identity of individual cell. Unlike Perturb-seq, CROP-seq and CRISP-seq sequence polyadenylated (polyA)-tailed gRNA transcripts that are under the control of RNAPII promoters. Therefore, no modification of single-cell gel beads is required for these two platforms. When CROP-seq compatible vectors are transduced into cells, two gRNA-expressing cassettes containing the same sequences are inserted into the host cell genome: one set for gene editing and the other set of polyA-tailed transcripts for gRNA assignment. In contrast, CRISP-seq compatible vectors contain unique guide indexes for each gRNA, which can be transcribed by RNAPII. gRNA assignment in CRISP-seq is achieved by sequencing unique guide indexes. These scCRISPR screen platforms have been successfully applied to outline regulatory networks of biological pathways (14,16), identify autism risk genes, (17) and map regulator DNA segments for gene transcription (18).

Currently, scCRISPR screen technology is still in an early developmental stage and is mainly applied in screens involving one cell type. However, more than one type of cell is usually required to address immune-related questions. Therefore, performance evaluation and experimental optimization are needed to implement this new technology into immune-related screens. In our previous studies, we employed tumor cell lines and their paired tumor-reactive T cells to functionally interrogate the roles of tumor intrinsic factors in immune resistance using genetic screening approaches (10,19). Our screens have identified a series of tumor intrinsic factors with the potential to modulate antitumor immune responses. Among these identified factors, *in vitro* and *in vivo* studies confirmed that the expression levels of *PRMT1* (10), *RIPK1* (10) or *AXL* (19) in tumor cells correlate with tumor responses to immune attacks. Bulk RNA-seq has been used to characterize changes in transcriptional profiles resulting from targeting these factors in tumor cells. By leveraging the resources generated in our previous studies, we seek to compare the performance of different scCRISPR screen platforms in determining underlying mechanisms of tumor intrinsic immune regulation and to optimize experimental designs and data analysis in scCRISPR immune screens.

In this study, we focused on the comparison and optimization of the Perturb-seq and CROP-seq due to their ease to construct compatible gRNA-expressing vectors. We knocked out the expression of *Prmt1, Ripk1* or *Axl* in a murine MC38 tumor cell line by gRNA-expressing vectors compatible with either Perturb-seq or CROP-seq. We evaluated the effects of each perturbation on gene expression profile and responses to *in vitro* T cell killing and *in vivo* anti-PD-1 treatment at the single-cell level. Our results demonstrate that scCRISPR screen platforms can efficiently characterize gene expression profiles and perturbation effects present in individual cells during both *in vitro* and *in vivo* immune-related screen conditions. Transcription profiles defined by our optimized scCRISPR screen platform recapitulate the perturbation effects characterized previously by bulk RNA-seq. Results from scCRISPR immune screens also predict transcriptional phenotype associated with clinical responses to cancer immunotherapy. More importantly, scCRISPR screen platforms enable us to determine the heterogeneous changes of gene expression in *Prmt1, Ripk1* or *Axl* knockout (KO) tumor cells in the presence of immune attack, which cannot be easily assessed by bulk RNA-seq. Taken together, scCRISPR screen technologies provide scalable and reliable platforms to validate identified tumor intrinsic immune factors in genome-wide screens and elucidate their working mechanisms.

## MATERIALS AND METHODS

### Cell lines and mice

MC38 and HEK293T cells were obtained from the National Cancer Institute (NCI) and the American Type Culture Collection (ATCC), respectively. All cells were cultured in RPMI-1640 medium with 10% heated-inactivated fetal bovine serum (FBS; #S11150, R&D System, Minneapolis, MN) and 100 μg/ml of Normocin (#ant-nr-1, InvivoGen, San Diego, CA). Gp100 and Cas9-expressing MC38 cells (MC38/GC) generated in our previous study (10) were cultured under the treatment of 10 μg/ml of blasticidin (#A1113903, Gibco, Carlsbad, CA) with the culture media described above. When necessary, culture medium was supplemented with 1 mM sodium pyruvate (#11360-070), 2 mM glutamax (#35050-061), 1× non-essential amino acids (#11400-050) from Gibco. All cell lines were authenticated by short tandem repeat fingerprinting or the expression of tagged markers used for genetic modification. The mycoplasma detection kit (#13100-01, SouthernBiotech, Birmingham, AL) was used to routinely monitor for mycoplasma contamination of cultured cells. The maximum length of time of *in vitro* cell culture between thawing and use in the described experiments was two weeks.

Pmel-1 TCR/Thy1.1 (Pmel) transgenic mice were provided by Dr Nicholas Restifo (NCI) and in-house bred. C57BL/6 mice were purchased from the Charles River Frederick Research Model Facility. All mice were maintained in a specific pathogen-free barrier facility at the University of Houston. Mice were handled following protocols approved by Institutional Animal Care and Use Committees.

### Generation of gp100-specific T cells

Gp100-specific T cells used in *in vitro* killing assays were generated as previously described (10). Briefly, splenocytes were isolated from Pmel mice and cultured in complete RPMI-1640 media (10% FBS, 20 mM HEPES, 1 mM sodium pyruvate, 0.05 mM 2-mercaptoethanol, 2 mM l-glutamine and 100 μg/ml of Normocin) supplied with 500 IU/ml interleukin-2 (rhIL-2; Prometheus Laboratories, NDC Code 65483-116-07, San Diego, CA) and 0.3 μg/ml anti-mouse CD3 (#555273, BD Biosciences, San Jose, CA) for 24 h. After stimulation, cells were maintained in the culture medium with 500 IU/ml IL-2 for at least 6 days. After 6- to 10-day culture, Pmel T cells were used in described experiments.

### Construction of scCRISPR-compatible lentiviral vectors for gRNA expression.

To construct Perturb-seq compatible vectors, the lentiviral gRNA-expressing vector, pKLV2-U6gRNA5(BbsI)-PGKpuro2ABFP-W (#67974, Addgene, Watertown, MA), was modified to insert different capture sequences at varied locations, as described in the Chromium Single Cell 3' Reagent Kits User Guide (v3.1 Chemistry) with Feature Barcoding technology for CRISPR Screening (10× Genomics, Pleasanton, CA). The backbone vector and four pairs of fully synthesized double-strand (ds) DNA fragments (Twist Bioscience, San Francisco, CA), whose forward sequences are listed in Supplementary Table S1, were digested by MluI-HF and BamHI-HF (#R3198S and #R3136S, New England Biolabs, Ipswich, MA) to expose cohesive ends. The linearized backbone vector was conjugated with each dsDNA fragment by T4 DNA ligase (#M0202S, New England Biolabs) at 16°C overnight. Four modified vectors named pKLV2-Perturb-type I, type II, type III, and type IV were constructed. Then, pKLV2-Perturb vectors were digested with BbsI-HF (#R3539S, New England Biolabs) and conjugated with dsDNA fragments encoding gene-specific gRNAs or non-targeting gRNAs by T4 DNA ligase at 16°C overnight. For CROP-seq compatible vectors for gRNA expression, the lentiviral vector, CROPseq-Guide-Puro (#86708, Addgene), was digested with BsmBI-v2 (#R0739S, New England Biolabs) and then conjugated with dsDNA fragments as previously described. All protospacer sequences of gene-specific gRNAs targeting murine *Prmt1, Ripk1* and *Axl* together with two individual non-targeting gRNAs (NC1 and NC2) are listed in Supplementary Table S2. All constructed vectors were validated by Sanger sequencing.

### Establishment of genetically modified tumor cell lines

To generate lentiviral supernatants, HEK293T cells were seeded 16 h prior and followed by transfection with gRNA-expression vectors along with lentiviral packaging plasmids, pCMV-VSV-G and psPAX2 (#8454 and #12260, Addgene) by the jetPRIME transfection reagent (#101000046, VWR, Radnor, PA) according to the manufacturer's protocol. Viral supernatants were collected 72 h post-transfection and filtered by a 0.45 μm PVDF Syringe Filter Unit (#SLHV033NK, Millipore-Sigma, Burlington, MA) to remove cell debris. Designated titers of lentivirus were used to infect MC38/GC cells in the presence of 8 μg/ml hexadimethrine bromide (#107689, Sigma-Aldrich, St Louis, MO). After 48 hours transduction, cells were selected with a completed growth medium in the presence of 2 μg/ml of puromycin (#A1113803, Gibco) to establish stable cell lines. For each scCRISPR screen platform, five different types of modified MC38/GC lines including gNC1, gNC2, gPrmt1, gRipk1 and gAxl cell lines were individually generated by using identical transduction conditions.

### *In vitro* 2CT-scCRISPR screen

Equal numbers of five types of modified MC38/GC cell lines were pooled together. Total 10 × 10^6^ pooled MC38/GC cells were pre-seeded into T150 cell culture dishes for 8 h, then co-incubated with 10 × 10^6^ cultured Pmel T cells in presence of 300 IU/ml IL-2 for additional 16 h. Tumor cells in a medium with 300 IU/ml IL-2 were served as the control group. After T cell co-culture, non-adhesive cells were removed by repeated washing with pre-warmed PBS. Adherent cells were trypsinized, resuspended into PBS supplied with 2% FBS, and filtered with a 70 μM cell strainer. In the Perturb-seq screen, single-cell suspensions were directly applied for scRNA-seq preparation. In the CROP-seq screen, single-cell suspensions were stained with mCD45-PE (#553081, BD bioscience) for 30 min and followed by sorting viable CD45^−^ tumor cells using the BD FACS Melody™ Cell Sorter. Viable cells were gated based on the forward scatter and side scatter. Sorted tumor cells were applied for scRNA-seq sample preparation.

### *In vivo* ICB-scCRISPR

Equal numbers of five types of modified MC38/GC cell lines were pooled together before tumor inoculation. 5 × 10^5^ pooled gRNA-expressing MC38/gp100 tumor cells were subcutaneously injected into each C57/BL6 mouse. Seven days after tumor inoculation, tumor-bearing mice were intraperitoneally administrated with either anti-PD-1 (#135247, BioLegend, San Diego, CA) or control antibody (#BE0085, Bioxcell, Lebanin, NH) at the dose of 100 μg/dose every 2 days. Five mice for each treatment group were included. Tumor samples were collected on day 7 or day 14 after antibody treatment in the Perturb-seq and CROP-seq screens, respectively. Tumor sizes in all experimental mice were monitored by measuring the perpendicular diameters of the tumors. 100μg of a tumor tissue sample from each mouse was harvested, cut into small pieces, and incubated in an RPMI-1640 medium containing 1 mg/ml collagenase (#C0130, Sigma-Aldrich) and 100 μg/ml hyaluronidase (H6254, Sigma-Aldrich) at 37°C for 60 min. After enzymatic digestion, tumor tissues were manually dissociated to generate single-cell suspensions. Equal amounts of single-cell suspensions from individual tumor tissues in the same group were pooled together. Tumor cells were sorted based on the expression of CD45 as described previously. All experiments were carried out in a blinded, randomized fashion.

### gRNA expression determined by real-time PCR

To determine the impact of transduction efficiency on gRNA expression levels, MC38/GC cells were transduced with varying amounts of lentivirus expressing scCRISPR-compatible vectors to achieve a differing multiplicity of infection (MOI; Low MOI = 0.2 versus High MOI = 1). Transduced tumor cells were selected by puromycin as previously described. Total RNAs were isolated from modified MC38/GC cells by TRIzol (#15596026, Invitrogen, Waltham, MA) according to the manufacturer's instructions. For RNA samples from cells expressing pKLV2-Perturb vectors, cDNA synthesis was performed in the first step using total RNA (500ng) with random primers and iScript Reverse Transcription Supermix (#1708840, Bio-Rad, Hercules, CA). For RNA samples from cells expressing CROP-seq vectors, cDNA synthesis was performed in the first step using total RNA (500 ng) with oligo(dT) and SuperScript™ III First-Strand Synthesis System (#18080-051, Invitrogen). Quantitative real-time PCR (qRT-PCR) was used to determine levels of gRNAs, *Actb* mRNA, and *Ripk1* mRNA. Triplicated PCR reactions by using SsoAdvanced Universal SYBR Green Supermix (#1725274, Bio-Rad) were run on an ABI 7500 (Applied Biosystems, Bedford, MA) according to the manufacturer's protocol. The gene expression level was normalized with the related genes and the average 2^−ΔΔCt^ value was calculated correspondingly. The fold changes relative to respective controls were determined. The primer information is provided in Supplementary Table S3.

### Genomic DNA isolation and gRNA distribution detection by next-generation sequencing (NGS)

The gRNA distribution in genomic DNA samples from tumor cells was evaluated by NGS as previously described (10). Briefly, genomic DNA from tumor cells was extracted by using the QIAamp DNA Mini Kit (#51304, Qiagen, Germantown, MD) according to the manufacturer's protocol. DNA concentrations were quantified with a Nanodrop (Invitrogen). The gRNA fragments were amplified and barcoded with adaptation by nested PCR. The PCR primers are listed in Supplementary Table S3. The PCR products were purified by using the NucleoSpin Gel and PCR Clean-up Kit (#740609.50, Macherey-Nagel, Allentown, PA), then sequenced by using a NextSeq 550 (Illumina, San Diego, CA) at the Advanced Technology Genomics Core of The University of Texas MD Anderson Cancer Center (MDACC).

### Single-cell partitioning for scRNA-seq

The single-cell partitioning method provided by 10× Genomics was used for both the Perturb-seq and CROP-seq platforms. Single-cell suspensions at a concentration of 1000 live cells/μl were prepared. Around 3000–10 000 cells were captured per lane of a 10× Chromium device by using Single Cell 3′ Solution reagents (10× Genomics). In Brief, single cells, RT reagents, single-cell gel beads, and oil were loaded on a Chromium controller (10× Genomics) to generate single-cell Gel Beads-In-Emulsions (GEMs) where full-length cDNAs were synthesized and barcoded for every single cell. Subsequently, the GEMs were broken and cDNAs from every single cell were pooled for further process.

### 3′ Gene expression (GEX) library and gRNA library preparation

For the Perturb-Seq platform, 3′ GEX and gRNA libraries were prepared using the Chromium Next GEM Single Cell 3′ Reagent Kits V3.1 with Feature Barcoding technology for CRISPR Screening (#PN-1000128,10× Genomics). The construction of the 3′GEX library and gRNA library was performed as described in the Chromium Single Cell 3' Reagent Kits User Guide (v3.1 Chemistry) with Feature Barcoding technology for CRISPR Screening (Rev_D). The constructed libraries were sequenced by a NextSeq 500 (Illumina) in the Seq-N-Edit Core at the University of Houston. For the CROP-Seq platform, a 3′ GEX library was constructed by using the Chromium Next GEM Single Cell 3' Kit v3.1 (#PN-1000147, 10× Genomics), according to the instructions provided in the Chromium Single Cell 3' Reagent Kits User Guide (v2 Chemistry) (Rev_F). To enrich polyA-tailed gRNA transcripts in 3′GEX libraries, a three-round hemi-nested PCR reaction was performed as previously described (20). Briefly, in the first round of nested PCR reactions, 20ng of full-length cDNAs were amplified by the KAPA HiFi system (#07958935001, Roche, Pleasanton, CA) at 65°C for annealing temperature. In the second round of PCR reactions, 1/25 of purified first-round PCR products were used for amplification (65°C for annealing temperature). In the final round of PCR reactions, 1/25th of purified second-round PCR products were used for amplification at 72°C for annealing temperature. The PCR products from final rounds were purified and applied for a dual size selection using 0.6–1x Ampure beads (#A63880, Beckman Coulter, Brea, CA). All primers to enrich gRNA transcripts are listed in Supplementary Table S3. The constructed libraries were sequenced by using a Novaseq 6000 (Illumina) in the Single Cell Genomics Core at Baylor College of Medicine.

### Immunoblot analysis

Proteins were extracted by lysed tumor cells using 2× Laemmli Lysis Buffer (#1610737, Bio-Rad). The western blot analysis was used to determine expression levels of proteins of interest. The protein bands were detected by ECL Plus (#WBKLS0500, Millipore-Sigma) using the ChemiDoc Imaging System (Bio-Rad). Anti- PRMT1 antibody (#2449) and anti-RIPK1 antibody (#3493) were purchased from the Cell Signaling Technology and anti-AXL antibody (#AF854) was purchased from the R&D Systems.

### Statistical and bioinformatics analyses for scRNA-seq

All raw sequence data generated from scRNA-seq were firstly processed by the 10× Genomics Cell Ranger pipeline. Briefly, the sequencing output data (BCL files) were demultiplexed, converted into FASTQ files, and then aligned to the mm10-3.0.0 reference genome and the protospacer sequences of gRNAs to produce count matrices for mRNA and gRNAs. The count matrices were imported into Seurat (4.0.0) in R (4.0.4) for further filtering, normalization, log-transform, scaling, dimensional reduction by Principal Component Analysis (PCA), and Uniform Manifold Approximation and Projection (UMAP) for clustering. Summary statistics of data from each scCRISPR screen are reported in Supplementary Table S4.

To compare the transcriptional profiles determined by scRNA-seq and bulk RNA-seq, we created a pseudo-bulk profile using log-transformed average scRNA-seq counts. The results were compared with log_2_ counts per million reads mapped (CPM) obtained by bulk RNA-seq. Pearson correlation was used to assess the degree of concordance. Different constants ($c$) were tested in the log_2_-transform log_2_(count + $c$) to identify suitable settings for relatively low read depth in scRNA-seq.

To generate the ‘supervised UMAP’, a random forest classifier trained on the gene expression profile with gRNAs as labels was utilized following the same procedure as previously described (14). Top 100 contributing genes of classification were used for generating the UMAP. To evaluate the difference of gene expression profiles between two groups of cells, out-of-bag scores serving as the accuracy measurement were calculated and listed in the Supplementary Figure S7. To perform a statistical test on the scores, labels were randomly assigned to cells by using the same process of accuracy calculation. The number of cells per label is maintained as it affects the accuracy. To obtain an empirical distribution of the scores, 1000-time repetitions were performed. Meanwhile, one-tailed *P*-value was calculated for the significant separation in gene expression profile.

Differential expression analyses (DEAs) were performed using limma-voom (3.44.3), one of the best-performing methods on scRNA-seq data (21). Genes expressed in fewer than 15% of the cells were filtered out to obtain a reasonable mean-variance trend and interaction effect of treatment (Pmel/anti-PD1). Gene perturbation (CRISPR KO) was accounted for in the analysis (GeneExpression ∼ treatment + CRISPR + interaction). The first two terms recognize differentially expressed genes (DEGs) as a result of treatment and CRISPR perturbation, respectively. The last interaction term identified genes that respond differently to treatment before and after CRISPR perturbation. Log_2_ fold-change, *P*-value and Benjamini–Hochberg false discovery rate adjusted *P*-value were reported.

### *k*-Nearest neighbors score

To determine if gene expression profiles of cells are clustered by perturbation, the *k*-nearest neighbors (k-NN) score (ranging from 0 to 1) was assessed as previously described (22), where a higher score indicates a more distinct clustering of a group of cells. The K-NN score of a cell is defined as the proportion of cells from the same group (i.e. gRNA) among the most similar transcriptomic profile. We take the average score over a group to measure the cohesiveness of a group. The expected score for a completely random transcriptomic profile is also calculated for reference. The scores are then averaged within each gRNA group and further among all groups. Under the null hypothesis of k-NN score, all cells are randomly distributed in the transcriptomic space. Thus, for each dataset, we randomly shuffled the cell categories 1000 times to generate a null distribution and calculated the k-NN score in the same manner. The *P*-value is then the normalized rank of the actual score.

### Statistical analysis of changes in proportions of cell clusters

We used *z*-statistics to determine the statistical significance and confidence interval of change in proportions for each cluster. For a cell group (e.g. gPrmt1) having ${n_1}$ treated cells and ${n_0}$ control cells, and proportions ${\hat p_1}$ and ${\hat p_0}$ of the cells show up in each cluster, respectively, the change in proportion (effect size) is ${\hat p_1} - {\hat p_0}$. To determine the significance of the change, we estimated the standard error by $\sqrt {\frac{{{{\hat p}_1}( {1 - {{\hat p}_1}} )}}{{{n_1}}} + \frac{{{{\hat p}_0}( {1 - {{\hat p}_0}} )}}{{{n_0}}}}$, and used two-tailed *z*-test to determine the *P*-value of $z = \frac{{( {{{\hat p}_1} - {{\hat p}_0}} )}}{{\sqrt {\frac{{{{\hat p}_1}( {1 - {{\hat p}_1}} )}}{{{n_1}}} + \frac{{{{\hat p}_0}( {1 - {{\hat p}_0}} )}}{{{n_0}}}} }}$. Statistical analysis of changes in proportions of cell clusters was performed as previously described (23). In addition, the 95% confidence interval of the change in proportion can be constructed as ${\hat p_1} - {\hat p_0} \pm z( {1 - \frac{\alpha }{2}} )\sqrt {\frac{{{{\hat p}_1}( {1 - {{\hat p}_1}} )}}{{{n_1}}} + \frac{{{{\hat p}_0}( {1 - {{\hat p}_0}} )}}{{{n_0}}}}$.

### Gene set enrichment analysis (GSEA)

GSEA (4.2.1) was performed to integrate scCRISPR screen data with datasets obtained from melanoma patients who received cancer immunotherapy (19,24). For each gene set, GSEA reports an enrichment score (ES) reflecting the degree to which a gene set is overrepresented at the top or the bottom of a list of genes ranked by correlation of the gene with the phenotype. GSEA walks down the ranked gene list, increasing a running-sum statistic when a gene is in the gene set and decreasing it when it is not. The magnitude of the increment depends on the correlation coefficients. The ES is the maximum/minimum running sum encountered. Genes leading to the ES are considered core enrichment. A positive ES indicates upregulation of the gene set and a negative ES indicates downregulation. To account for sizes of gene sets, GSEA uses the Normalized Enrichment Score (NES) defined as the quotient of ES and the average ES of permuted cell type labels. A *P*-value was also derived from the permutation. Benjamini-Hochberg method was used to obtain adjusted *P*-values.

### Ingenuity pathway analysis (IPA)

QIAGEN Ingenuity Pathway Analysis 8.5 (Version: 70750971) was used to select, annotate, and visualize genes by function and pathway. The DEGs with a cut-off of adjusted *P*-value at 0.25 were selected for the IPA analysis. IPA calculates a *P*-value for each gene set using a Right-Tailed Fisher's Exact Test to reflect the likelihood of the gene set and the DEGs being random. IPA analysis identified those canonical pathways differentially expressed (*P* < 0.05) between comparison groups.

### Over-representation analysis (ORA)

We used ‘enricher’ function in ‘clusterProfiler’ to perform the ORA, which does a hypergeometric test on genes with adjusted *P*-value < 0.25 in DE analysis. *P*-values for pathways are corrected by Benjamini–Hochberg (aka. FDR) approach. Gene ratio is calculated as *k*/*n*, where *k* is the number of DEG in a specific gene set/pathway (such as GOBP_REGULATION_OF_PEPTIDASE_ACTIVITY) and *n* is the total number of DEGs in the whole gene set collection (such as MSigDB C5).

### Gene ontology (GO) analysis

To determine the functional profiles of DEGs in the treatment-enriched clusters from *in vivo* scCRISPR screens, genes with *P*-value <0.05 and |Log_2_(Fold change)| >1 between treatment-enriched clusters and control-enriched clusters were selected for GO analysis using g:Profiler (version e106_eg53_p16_65fcd97) with g:SCS multiple testing correction method applying significance threshold of 0.05 (25). Two major sub-ontologies of GO_Biological Process and GO_Molecular Function terms were included in the GO analysis.

## RESULTS

### Establishment of *in vitro* and *in vivo* scCRISPR immune screens

**Figure 1.**
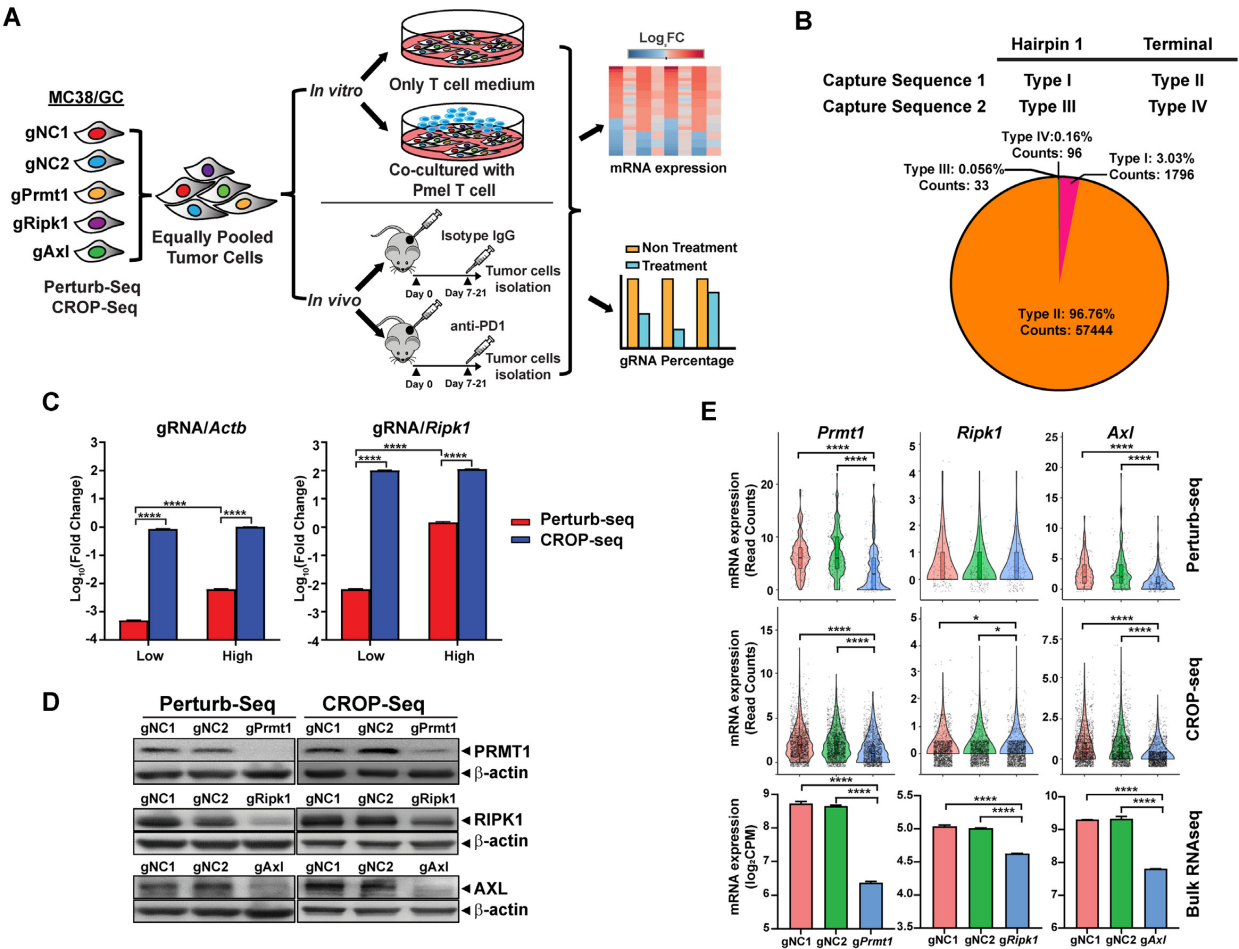
Establishment and vector optimization of scCRISPR immune screens. (**A**) A schematic diagram of the *in vitro* 2CT-scCRISPR screen and *in vivo* ICB-scCRISPR screen. Each set of the scCRISPR screen system includes five types of genetically modified MC38/GC cell lines expressing one of the gRNAs (gRNAs targeting *Prmt1*, *Ripk1* or *Axl* and two non-targeting gRNAs). Equal numbers of each type of gRNA-expressing tumor cells were pooled. For the *in vitro* 2CT-scCRISPR screen, pooled cells were co-cultured with gp100-specific T cells derived from Pmel mice for 16 h. Pooled cells without T cell co-culture were used as the control group. For the *in vivo* ICB-scCRISPR screen, pooled MC38/GC cells were used to challenge C57BL/6 mice. Mice bearing 7-day established tumor were intraperitoneally treated with anti-PD-1 (100 μg/dose, every 2 days). Tumor-bearing mice treated with an isotype control antibody were served as the control group. RNAs were isolated from tumor cells and sequenced to determine the transcriptional changes of each related genetic perturbation in the presence and absence of immune attack. (**B**) Identification of the optimal gRNA scaffold for the Perturb-seq platform. Four types of gRNA scaffolds varying in capture sequence and inserting location. A scRNA-seq analysis was used to evaluate the capture performance of each gRNA scaffold. The pie chart shows the percentages of cells expressing each type of gRNA in the pooled sample together with their total counts. (**C**) Evaluation of expression levels of transcripts containing gRNA sequences in the scCRISPR platforms. MC38/GC cells were transduced with either Perturb-seq-compatible vectors or CROP-seq-compatible vectors at different transduction conditions (Low MOI = 0.2; High MOI = 1). The expression levels of a house-keeping gene (*Actb*) and a moderately expressed gene (*Ripk1*) were also detected and used as control genes. Relative expression levels between gRNA transcripts and each control gene were calculated by using 2^−ΔΔCt^. (**D**) Evaluation of gene-specific knockout (KO) efficiency of different scCRISPR-compatible vectors at the protein level. Western blot analysis was used to determine the expression of target proteins in gRNA-expressing tumor cells. Cells expressing non-targeting gRNAs (gNC1 and gNC2) served as controls. (**E**) Evaluation of gene-specific knockout efficiency of different scCRISPR-compatible vectors at the transcriptional level. Perturb-seq, CROP-seq, and bulk RNA-seq analyses were used to determine the expression of target transcripts in gRNA-expressing tumor cells. Expression levels of *Prmt1*, *Ripk1* and *Axl* obtained from scCRISPR platforms were measured by read counts, and expression levels of *Prmt1*, *Ripk1*, and *Axl* obtained from bulk RNA-seq were measured by counts per million reads (CPM). Statistical significance between the gRNA groups and gNC groups was determined by one-way ANOVA with repeated measurements. *P< 0.05; ****P< 0.0001.

To implement Perturb-seq and CROP-seq in immune-related studies, we developed *in vitro* 2 cell type (CT)-scCRISPR screen and *in vivo* immune checkpoint blockade (ICB)-scCRISPR to explore tumor resistance mechanisms to cancer immunotherapy. Previously, we have genetically modified murine MC38 tumor cells (MC38/GC) to stably express both Cas9 and a tumor antigen, gp100. Based on our previous genetic screen studies, protospacer sequences of gRNAs targeting three validated immune regulators, namely *PRMT1*, *RIPK1*, and *AXL*, and two non-targeting gRNAs were selected to generate gRNA-expressing vectors compatible across the scCRISPR platforms. To better evaluate the potential of gRNA contamination caused by scRNA-seq sample preparation, five types of gRNA-expressing MC38/GC lines were individually generated for each scCRISPR screen platform and then pooled with an equal number of gRNA-expressing cells for immune screens. In such a way, we can rule out the possibility of cells containing multiple types of gRNAs which are generated by pooled viral transduction. Pooled tumor cells were subsequently followed by either *in vitro* gp100-specific-T-cell treatment or *in vivo* anti-PD-1 treatment (Figure 1A). gRNA reads from pooled tumor samples were used to assign the perturbation status of individual cell and to determine the abundance of each line in the pooled tumor samples, whereas mRNA reads were used to characterize transcriptional phenotypes caused by individual perturbations in the presence and absence of immune attack. Detailed distinct features between the Perturb-seq and CROP-seq platforms are summarized in Supplementary Figure S1.

Although the design of CROP-seq compatible gRNA-expressing vectors is unique, there are four different types of gRNA scaffold designs for the Perturb-seq platform (Supplementary Figure S2), varying in capture sequences (C1 and C2) and insert locations of capture sequences (hairpin 1 and terminal location). Here, we modified a commonly used lentiviral gRNA-expressing vector, pKLV2, which includes both a puromycin-resistant element and a fluorescent protein tag for selection. We added different protospacer sequences in pKLV2 based on four different types of designs (one unique sequence per type of design) and generated a set of tumor cell lines expressing these modified pKLV2 vectors. Then, equal numbers of each type of gRNA-expressing cells were pooled for gRNA library preparation. After sequencing the pooled tumor cell samples, we found that a majority of detected gRNA reads (>96%) are from the type II gRNA-expressing vector in which C1 was inserted at the termination location (Figure 1B), which is consistent with previously reported findings (26). Based on this result, the type II design was selected to construct gene-specific pKLV2 vectors in further Perturb-seq screens.

As detection efficiency by scRNA-seq positively correlates with RNA expression levels, we next compared expression levels of gRNAs in cells transduced with scCRISPR compatible vectors. The levels of gRNAs and polyA-tailed gRNA transcripts were measured in cells expressing a Perturb-seq compatible vector and in those expressing a CROP-seq compatible vector, respectively. Furthermore, the effect of viral transduction efficiency on gRNA expression was evaluated by using gRNA-expressing cell lines generated at high (1.0) and low (0.2) multiplicity of infection (MOI) conditions. For the Perturb-seq system, the expression level of gRNAs in high MOI-infected cells was comparable with the level of a moderately expressed gene (*Ripk1*), but a significant reduction of gRNA expression was found in low-MOI infected cells (Figure 1C). On the other hand, transduction efficiency had a limited impact on expression levels of polyA-tailed gRNAs transcribed from CROP-seq, which could achieve levels equivalent to that of a housekeeping gene, *Actb* (Figure 1C). Furthermore, we determined gene-specific knockout efficiency of scCRISPR compatible gRNA-expressing vectors. We constructed a set of scCRISPR compatible vectors encoding gRNAs targeting murine *Prmt1, Ripk1*, and *Axl* (one gRNA per target). Western blot analysis confirmed that both Perturb-seq and CROP-seq compatible vectors can significantly suppress the expression of target proteins in tumor cells when compared with cells expressing non-targeting gRNAs (Figure 1D). Similar to the bulk RNA-seq results, both scCRISPR platforms successfully detected gene-specific perturbation in cells with gRNAs targeting *Prmt1* (gPrmt1) or *Axl* (gAxl; Figure 1E). However, inhibition of *Ripk1* by the *Ripk1*-specific gRNA (gRipk1) induced moderate change (around 20%), as shown in the bulk RNA-seq result. We only observed a significant reduction of *Ripk1* expression in gRipk1-expressing cells by the CROP-seq system, not by the Perturb-seq system. These results suggest that both scCRISPR-compatible vectors can achieve a similar level of gene-specific inactivation as standard gRNA-expressing vectors, and CROP-seq vectors could result in higher expression of polyA-tailed gRNAs than the level of gRNAs expressed by Perturb-seq vectors. Moreover, the CROP-seq platform could be more sensitive to detecting moderate differences in gene expression than the Perturb-seq platform.

### Optimization of scCRISPR immune screens

Our previous studies identified three tumor intrinsic factors, namely *PRMT1* (10), *RIPK1* (10) and *AXL* (19), as immune regulators. Targeting each of these factors had been reported to alter tumor responses to *in vitro* T-cell killing and/or *in vivo* anti-PD-1 treatment. We leveraged these results to evaluate and optimize the performance of immune-related scCRISPR screens. As described above, sets of MC38/GC cell lines expressing g*Prmt1*, g*Ripk1*, g*Axl*, or non-targeting gRNAs (gNC1/gNC2) were generated for the Perturb-seq and CROP-seq platforms. Four types of independent immune screens using pooled gRNA-expressing MC38/GC tumors were performed, namely *in vitro*-Perturb, *in vivo*-Perturb, *in vitro*-CROP, and *in vivo*-CROP. The technical specifications of these immune screens are summarized in Supplementary Table S4. The low yield rate (≈30% of input cells) of cells with high quality data based on Cell Ranger analysis was observed in our first scCRISPR immune screen, *in vitro*-Perturb. Furthermore, no treatment effect of using 7-day treated samples was found in our first *in vivo* immune screen. Therefore, we incorporated sorting for viable tumor cells and extended the duration of *in vivo* treatments in the optimized experimental protocols. The numbers of cells with both mRNA and gRNA reads in the remaining screens, particularly in CROP-seq screens, were very close to input numbers. It implied that cell sorting before encapsulation could significantly improve cell recovery rate.

Next, we set three criteria to remove low-quality cells from scCRISPR immune screens. First, even with cell sorting, a small portion of non-tumor cells could be detected in pooled samples from both *in vitro* and *in vivo* immune screens, and clustering by the Leiden algorithm could easily filter these non-tumor cells (Supplementary Figure S3A). Next, we also observed a negative association between the percentage of mitochondrial-encoded genes (mtDNA) reads and the total count of mRNA reads in individual cells (Supplementary Figure S3B), consistent with previous reports indicating that high mtDNA percentage is one of the biological features of low-quality cells (27). Finally, to appropriately assign gRNA to individual cells, we varied the threshold of gRNA counts per cell to select high-quality cells and determined the accuracy and efficiency of each threshold. As shown in Supplementary Figure S3C, at least 3 gRNA reads per cell could be obtained in >95% of sequenced cells. Furthermore, the percentages of cells with more than one type of gRNAs achieve a plateau for thresholds above 3. Based on these results, the criteria of high-quality gRNA-expressing cells used for further analysis are (i) within the cluster(s) for tumor cells; (ii) percentage of mtDNA reads <7.5%; (iii) count of gRNAs ≥3.

Moreover, we optimized the bioinformatics analysis pipeline of scCRISPR platforms by comparing transcriptional profiles of tumor cells detected by scRNA-seq and bulk RNA-seq. Untreated tumor samples from the control group among *in vitro*-CROP screens were sequenced at two sequencing conditions. The deep sequencing condition provided around 4MB/per cell of raw data. However, the shallow sequencing condition at 1.2MB/per cell is a more feasible experiment condition for scCRISPR screens containing >100 different gRNAs. We found that the data transformation method has a limited effect on the correlation between scRNA-seq results and bulk RNA-seq results at the deep sequencing condition (Supplementary Figure S3D). However, the log_2_(*X* + 0.001) transformation of scRNA-seq data from a shallow sequencing condition can dramatically increase the correlation coefficient (from 0.540 to 0.736; Supplementary Figure S3D).

### Performance evaluation of scCRISPR immune screens

**Figure 2.**
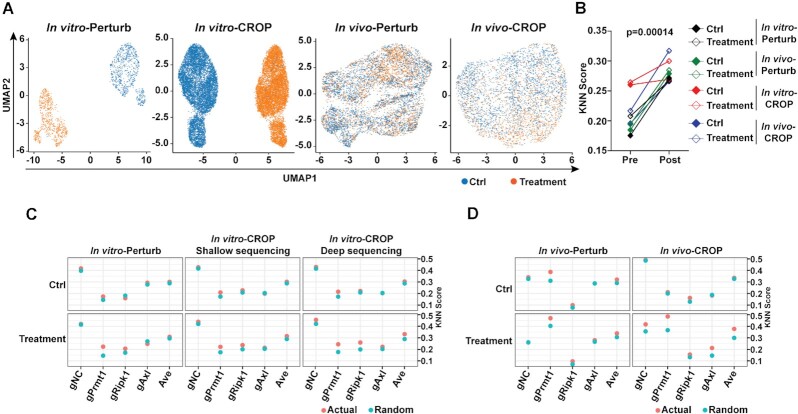
Performance evaluation and optimization of bioinformatics analysis pipeline of scCRISPR immune screens. (**A**) Treatment effects of *in vitro* and *in vivo* immune screens were revealed by Perturb-seq and CROP-seq. Uniform Manifold Approximation and Projection (UMAP) plots show clusters of cells in the control group (blue dots) and cells in the treatment group (orange dots). (**B**) Comparison of k-NN scores using raw scCRISPR results and results processed by the optimized bioinformatics analysis pipeline. (**C, D**) Dot plots showing k-NN scores at different types of gRNA-expressing cells obtained from *in vitro* 2CT-scCRISPR screens (C) and *in vivo* ICB-scCRISPR screens (D). Presumptive k-NN scores with random clustering were calculated and labeled with ‘Random’ (blue dots). Actual k-NN scores observed in scCRISPR immune screens were labeled with ‘Actual’ (orange dots).

Although we modified the experimental conditions in reported scCRISPR immune screens, we only included limited diversity of gRNAs (five types) in pooled samples and sequenced abundant cell numbers in these screens (Supplementary Table S4). Therefore, we still obtained sufficient data to fairly compare the sequencing performance of 3′-end Perturb-seq and CROP-seq at the baseline condition. To evaluate the capability of scCRISPR in determining perturbation phenotypes, we first sought to define the effects of *in vitro* T cell killing and *in vivo* anti-PD-1 treatment through unsupervised clustering based on transcriptome similarity among tumor cells with or without treatment. Both Perturb-seq and CROP-seq successfully distinguished cells treated with T-cell from control cells, while the segregation between control cells and treated cells in *in vivo* immune screens is minimal (Figure 2A). *k*-NN analysis is one of the most widely used methods to measure if cells in a group (i.e. gNC, gPrmt1, etc.) tend to have similar transcriptomic profiles. The results showed that our data filtering and transformation consistently improve the clustering of tumor cells based on gRNA identity in all experimental groups (Figure 2B). Moreover, we compared actual k-NN scores of gRNA-expressing cells with corresponding *k*-NN scores caused by random distribution. In majority of screens at the baseline condition, the actual k-NN scores of each type of gRNA-expressing cells are comparable with random k-NN scores except for gPrmt1-expressing cells, which suggested that significant alterations had already occurred at the transcription level after *Prmt1* knocking down (Figure 2C and D, Supplementary Table S5). However, cells expressing gene-specific gRNAs in treatment groups tend to group together. The actual *k*-NN scores of gPrmt1 cells displayed the most significant difference when compared with their corresponding random *k*-NN scores after *in vitro* or *in vivo* treatment (Figure 2C and D, Supplementary Table S5). These results imply that the genetic perturbation could result in additional transcriptional changes in the presence of immune attack, which cannot be easily detected by conventional bulk RNA-seq.

**Figure 3.**
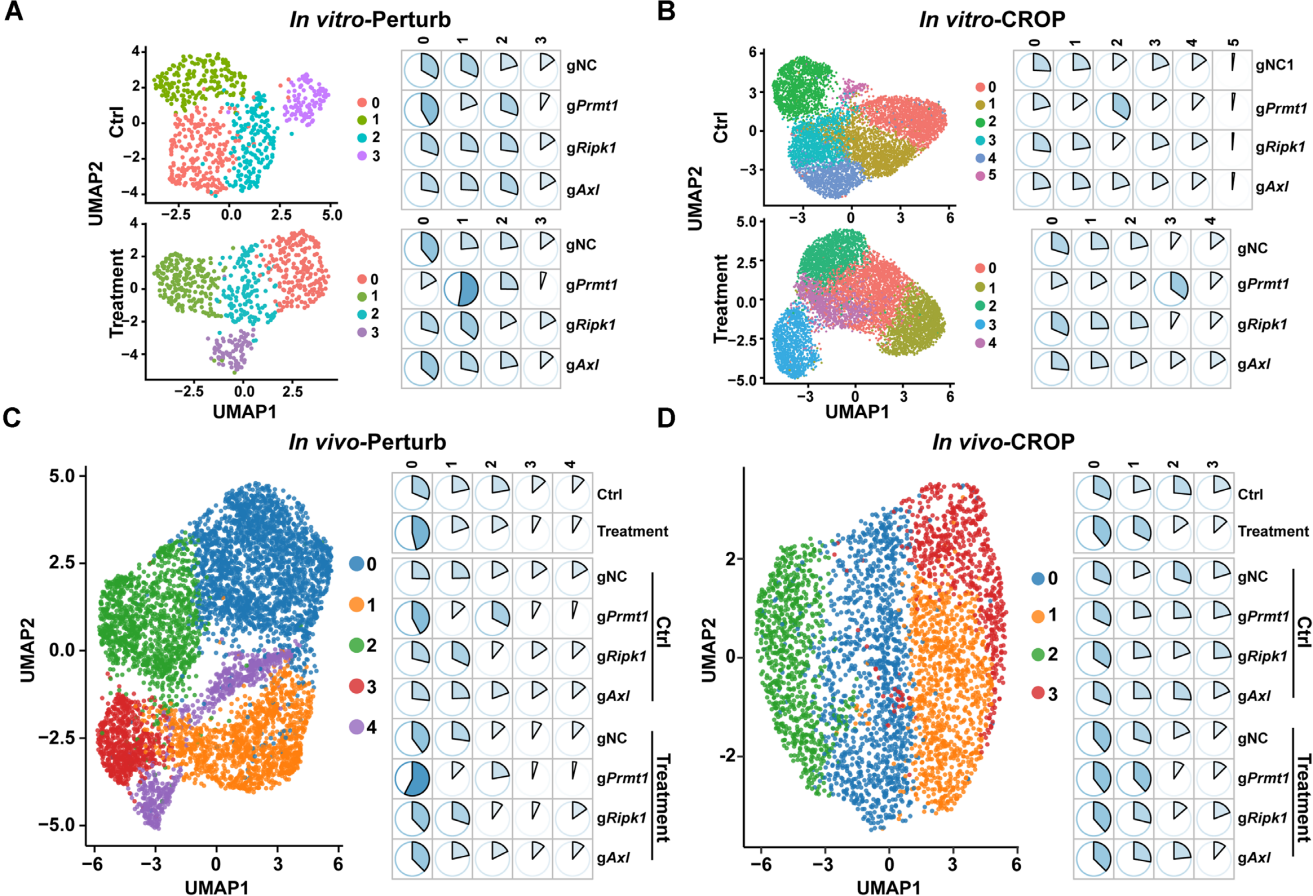
The scCRISPR immune screens reveal gene-specific effects of perturbing tumor intrinsic immune factors in response to *in vitro* T-cell killing and *in vivo* anti-PD-1 treatment. (**A, B**) The clustering and distribution of gRNA-expressing cells in response to *in vitro* T-cell treatment by the Perturb-seq platform (A) and the CROP-seq platform (B). For *in vitro* 2CT-scCRISPR screens, pooled gRNA-expressing MC38/GC cells were treated with tumor-reactive T cells for 16 h. Tumor cells receiving mock treatment were served as controls. (**C, D**) The clustering and distribution for gRNA-expressing cells in response to *in vivo* anti-PD-1 treatment by the Perturb-seq platform (C) and the CROP-seq platform (D). For *in vivo* ICB-scCRISPR screens, pooled gRNA-expressing MC38/GC cells were subcutaneously challenged into C57BL/6 mice. Tumor-bearing mice were treated with either Isotype antibody control group) or anti-PD-1 (treatment group) for 7 days (Perturb-seq) or 14 days (CROP-seq). gRNA transcripts and mRNA transcripts from treated tumor cells were analyzed by indicated scCRISPR platform. UMAP plots (left panels of each figure) and pie charts (right panels of each figure) were used to show cell clusters and percentages of variant clusters in indicated populations, respectively. In the listed pie charts, sum of each row equals 100%.

The unsupervised clusters of sequenced cells based on their transcriptional profiles are shown in Figure 3A–D, Supplementary Figure S4A and B. To better characterize the features of clusters showing different abundancy between the control and treatment groups, we selected one cluster enriched in the control groups (*In vivo*-Perturb: Cluster 1; *In vivo*-CROP: Cluster 2) and one cluster enriched in the treatment groups (Cluster 0 in both screens). Two-cluster comparisons using Wilcoxon test were performed to identify DEGs (Supplementary Figures S5A and B). The gene ontology (GO) analysis using identified DEGs were performed as previous described. It showed that the clusters enriched in the treatment group display upregulation of genes mainly involved in the cell cycle regulation (Supplementary Figure S5C) and downregulation of genes mainly classified as the extracellular matrix structural constituent (Supplementary Figure S5D).

Furthermore, we observed that the distribution pattern of gPrmt1 cells in the treatment groups was different from cells expressing non-targeting gRNAs regardless of scCRISPR screen platforms (Figure 3A–D). Particularly, as shown in the CROP-seq *in vivo* screen (Figure 3D), we observed that anti-PD-1 treatment increased the abundance of clusters 0 and 1, and decreased the abundance of clusters 2 and 3, suggesting that the first two clusters were enriched for PD-1 responding cells and other two clusters were enriched for PD-1 non-responding cells. The percentages in each defined cluster among different types of gRNA-expressing cells were comparable within the control group (Figure 3D). However, after anti-PD-1 treatment, more gPrmt1 cells were in the PD-1 responding population (cluster 0 and 1, *P* = 0.014 and 1 × 10^−7^, 95% CI [0.02, 0.14] and [0.09, 0.21]) and less gPrmt1 cells are in the PD-1 non-responding population (clusters 2 and 3, *P* = 3 × 10^−9^ and 3 × 10^−4^, 95% CI [−0.19, −0.9] and [−0.13, −0.04]) when compared with gNC cells (Figure 3D). These results are consistent with our conventional CRISPR screens showing that gPrmt1 cells are more sensitive to T cell killing (Supplementary Figure S6). Although both scCRISPR screen platforms could successfully reveal the distinct response of different gRNA-expressing cells to immune attacks based on their transcriptional profiles, scCRISPR screens failed to phenotype tumors cells by using percentages of each type of gRNA-expressing cells in pooled samples (Supplementary Figure S6). This limitation might be related to a small number of cells interrogated. Additionally, sorted cells were used in CROP-seq analysis, but not in Perturb-seq. By analyzing gRNA reads at the genomic level as described in conventional CRISPR screens, we found that treatment-related reduction of gPrmt1 intensity in the Perturb-seq screen using unsorted samples was around two folds higher than that in the CROP-seq screen using sorted samples (Supplementary Figure S6). This suggests that the sorting procedure in sample preparation might also restrain the power of gRNA distribution analysis in scCRISPR screens.

Finally, we performed supervised clustering analysis to better identify the distribution of single cells expressing a defined gRNA. By utilizing a random forest classifier training model as previously described(14), the accuracy and statistical evaluation of current results to predict each gene perturbation were calculated based on their transcriptional profiles from *in vitro*-CROP screen. Among three tested targets, our results achieved the best accuracy to predict gPrmt1 cells in the control and treatment groups (Supplementary Figure S7A). Furthermore, improved accuracy for gRipk1 cells was also observed in the treatment group when compared with the control group (Supplementary Figure S7A). Segregations between gNC cells and gPrmt1 cells were observed in the supervised UMAP plots (Supplementary Figure S7B). These results further support that the phenotypic changes caused by genetic perturbations can be more sensitively captured when immune attacks are present.

### Results from scCRISPR immune screens predict transcriptional phenotype associated with clinical responses to cancer immunotherapy

**Figure 4.**
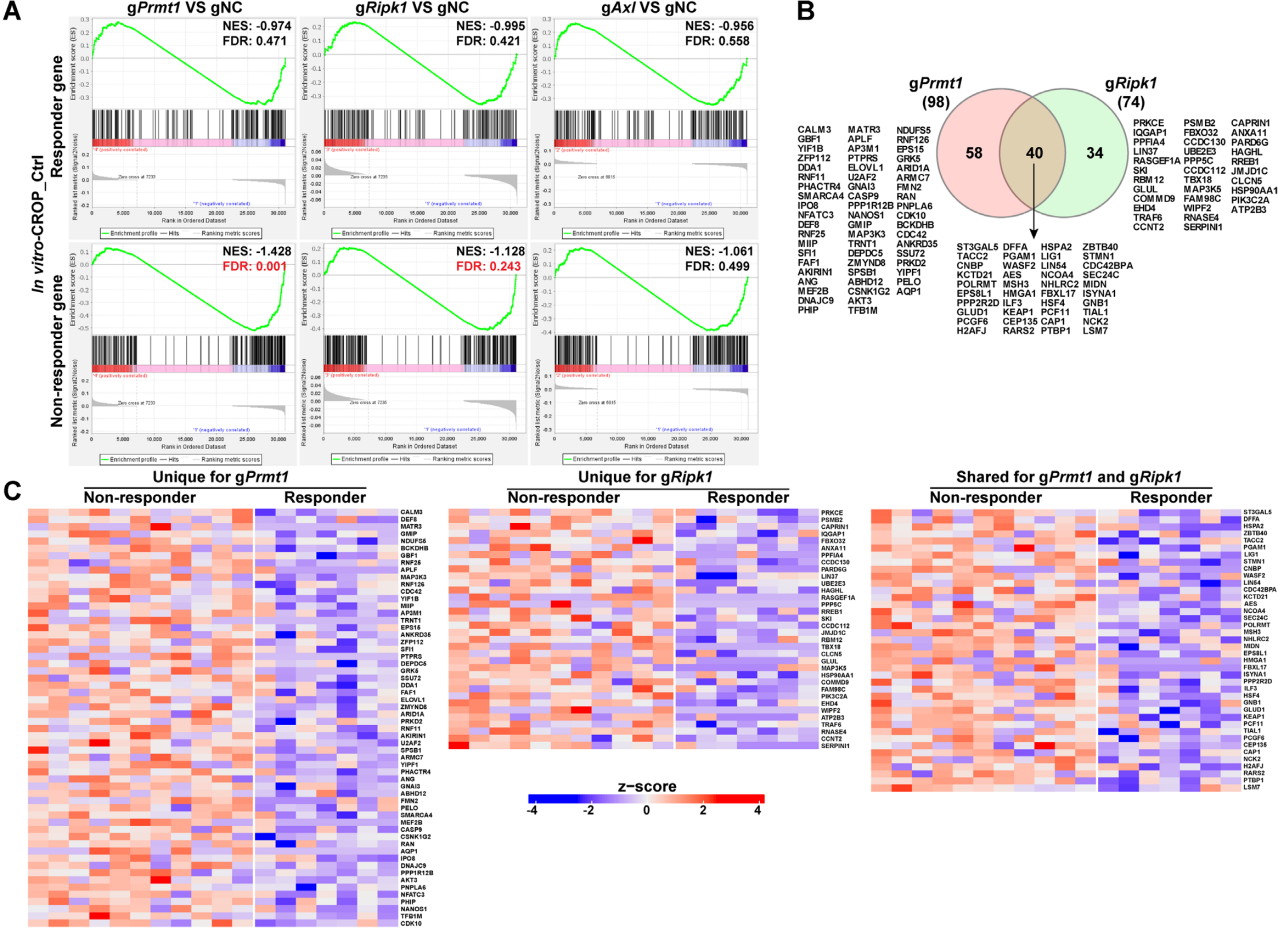
Inhibiting *Prmt1* or *Ripk1* downregulates expression levels of genes associated with poor clinical responses to adoptive T cell transfer (ACT). (**A**) Enrichment plots of gene sets associated with ACT responses in tumor samples with gene-specific perturbations. The mRNA expression profiles of tumor cell lines derived from pre-treatment tumor tissues in ACT-treated melanoma patients were previously determined. Based on clinical response to ACT (RECIST v.1.1), patients were stratified into the non-responders (with stable or progressive disease) and responders (with complete or partial response) groups. Differentially expressed genes (*P*< 0.05, by two-sided *t*-test) between tumor cells from non-responders and responders were identified. Upregulated genes and downregulated genes in non-responders were defined as the ‘ACT-Non-responder’ set and the ‘ACT-Responder’ set, respectively. Results from the control group in the *in vitro*-CROP screen were used to determine whether these two target sets are differentially expressed between MC38/GC cells with or without gene-specific-KO (gRNA versus gNC). NES, normalized enrichment score; FDR, false discovery rate. The value of FDR ≤0.25 was recognized as the statistical significance and labeled with red color. (**B**) A Venn diagram showing the number and list of overlapped and unique genes that are from the ACT-non-responder set and differentially expressed in gPrmt1 or gRipk1 cells. (**C**) Heatmaps demonstrating the relative expression levels of selected ACT-non-responder genes in melanoma lines derived from pre-treated tumor tissues. Genes that are differentially expressed in gPrmt1 or gRipk1 cells were selected for illustration.

Although the expression levels of gRNA transcripts captured by CROP-seq are higher than those by Perturb-seq, our results showed that both scCRISPR immune screen platforms can achieve gene-specific perturbation, and the transcriptional changes associated with genetic perturbations and treatments determined by these two platforms are highly correlated (Supplementary Figure S8). To further illustrate the clinical relevance of our immune screen results, we focused on using the CROP-seq results as representative data to examine whether targeting tumor intrinsic immune factors can alter the expression levels of DEGs between responders and non-responders to cancer immunotherapy. Our *In vitro* 2CT screens and *in vivo* ICB screens are designed to mimic adoptive T cell therapy (ACT) and anti-PD-1 treatment, respectively. Upregulated genes in responders and non-responders were identified from previously reported clinical datasets of ACT and anti-PD-1, and termed as responder genes and non-responder genes, respectively (19,24). By integrating *in vitro* 2CT screen data from the control group and the ACT dataset from melanoma lines derived from pre-treatment clinical samples, we observed that knocking out *Prmt1* or *Ripk1* can downregulate the expression of a set of genes associated with poor clinical outcomes in melanoma patients treated by ACT (Figure 4A). This result suggests that targeting these two factors might shift the transcriptional profiles of tumor cells towards those of the responders by downregulating the non-responder genes. However, genes differentially expressed in *Axl* KO tumor cells were not significantly enriched with the gene sets associated with ACT response (Figure 4A). Among non-responder genes that are downregulated in gPrmt1 or gRipk1 cells, around 50% of them are overlapped (Figure 4B), suggesting that the ACT-resistance mechanisms associated with *PRMT1* and *RIPK1* may be inflicted by different but related regulation pathways. The levels of non-responder genes modulated by depletion of PRMT1 and/or RIPK1 are further illustrated for ACT patient samples (Figure 4C). The fold changes in expression levels of ACT-non-responder genes in tumor cells with and without genetic perturbation are listed in Supplementary Table S6. Meanwhile, the expression differences between gPrmt1/ gRipk1/gAxl cells and gNC cells were demonstrated in the Supplementary Figure S9.

**Figure 5.**
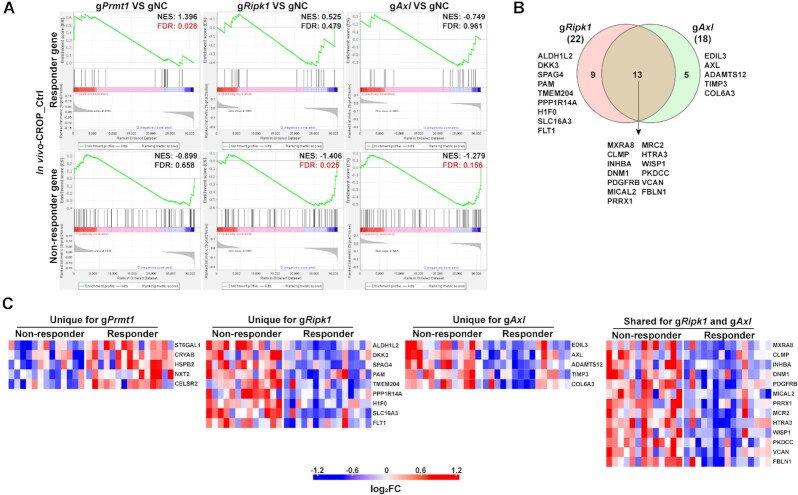
Inhibiting *Ripk1* or *Axl* downregulates expression levels of genes associated with poor clinical responses to anti-PD-1, while inhibiting *Prmt1* upregulates expression levels of genes associated with better clinical responses. (**A**) Enrichment plots of gene sets associated with anti-PD-1 responses in tumor samples with gene-specific perturbations. The mRNA expression profiles of pre-treatment tumor tissues from anti-PD-1 treated melanoma patients were previously determined. Based on clinical response to anti-PD-1 (RECIST v.1.1), patients were stratified into the non-responders (with stable or progressive disease) and responders (with complete or partial response) group. Differentially expressed genes (*P*< 0.05, by *t*-test) between tumor cells from non-responders and responders were identified. Upregulated genes and downregulated genes in non-responders were defined as the ‘ICB-non-responder’ set and the ‘ICB-responder’ set, respectively. Results from the control group in the *in vivo*-CROP screen were used to determine whether these two target sets are differentially expressed between MC38 cells with or without gene-specific-KO (gRNA versus gNC). The value of FDR ≤0.25 was recognized as the statistical significance and labeled with red color. (**B**) A Venn diagram showing the number and list of overlapped and unique genes that are from the ICB-non-responder set and differentially expressed in gRipk1 or gAxl cells. (**C**) Heatmaps showing relative expression levels of selected ICB-responder genes and ICB-non-responder genes in pre-treated melanoma tissues. Genes that are differentially expressed in gPrmt1, gRipk1 or gAxl cells were selected for illustration.

Next, we aimed to determine whether the expression of DEGs in KO tumor cells associates with clinical response to ICB in melanoma patients. By using a published dataset of pre-treatment biopsies from anti-PD-1 treated patients (24), we identified a set of genes whose expression levels are associated with treatment outcome. Then, we extracted the results of the control group from *in vivo*-CROP screen and performed GESA analysis using the responder gene set and non-responder gene set. As shown in Figure 5A, the responder genes are significantly upregulated in the gPrmt1 group, while non-responder genes are downregulated in both gRipk1 and gAxl groups, consistent with our hypothesis that these factors could be used as therapeutic targets to improve anti-PD-1 efficacy. Overlapped are most of the non-responder genes (around 60%) which are downregulated in gRipk1 and gAxl cells (Figure 5B). The fold changes in expression levels of ICB-non-responder genes in tumor cells with and without genetic perturbation are listed in Supplementary Table S7. Four of 13 overlapped markers (*MXRA8*, *CLMP, VCAN* and *FBLN1*) are involved in cell adhesion and tumor metastasis (28–33), suggesting that dysfunctions in cell adhesion pathways could lead to anti-PD-1 resistance associated by RIPK1 and AXL. Interestingly, among the sets of ICB-non-responder genes and ACT-non-responder genes that are potentially controlled by these three immune regulators (Figures 4C and 5C), there are no overlapped molecules, implying that the effects of inhibition of *PRMT1, RIPK1* and *AXL* on controlling primary responses to immunotherapy might not be shared across different types of treatments.

### scCRISPR immune screens reveal the interaction relationship between targeting tumor intrinsic immune factors and T cell-mediated antitumor immune response

**Figure 6.**
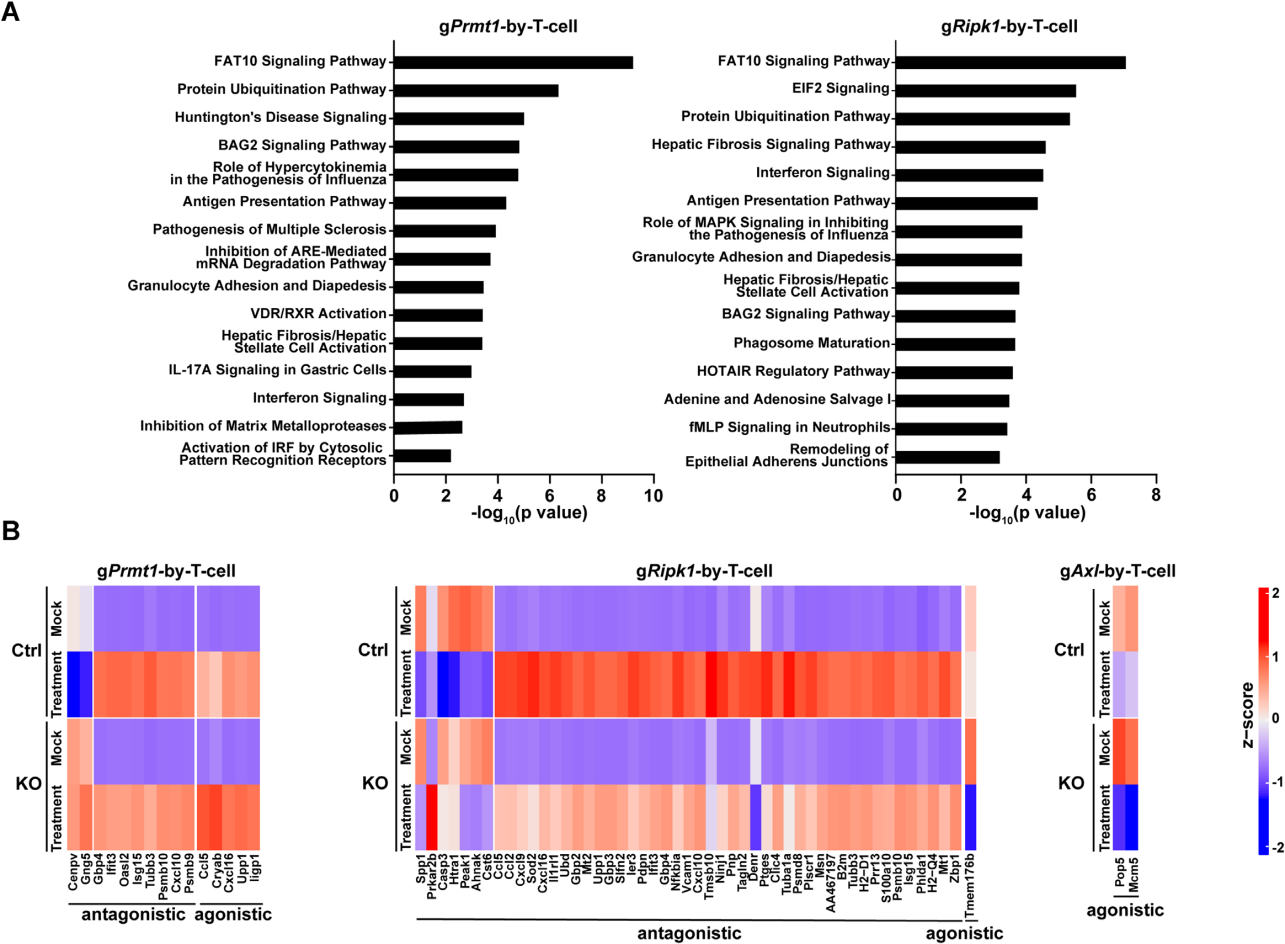
The interaction effects of gene-specific perturbations and *in vitro* T cell killing. (**A**) Ingenuity Pathway Analysis (IPA) of genes whose expression levels in tumor cells are modulated by KO-by-T-cell interaction. Statistical significances of the interaction effects between genetic perturbation and *in vitro* T cell treatment in the *in vitro*-CROP screen were determined. Genes in which the interaction effect has adjusted *P* <0.25 were defined as ‘interaction’ genes and selected for IPA. 41 of 1877 total detected genes and 240 of 1859 total detected genes were identified as genes with interaction effect in gPRMT1 and gRipk1 cells, respectively. The top 15 canonical pathways displaying statistically significant interaction effects in each genetic perturbation were listed. (**B**) Heatmaps showing the relative expression levels of top ‘interaction’ genes in *Prmt1*, *Ripk1*, and *Axl* KO tumor cells with or without T cell treatment. Up to the top 50 ‘interaction’ genes with the highest fold change of the interaction effect and together with adjusted *P*-value <0.1 were listed in the heatmap.

**Figure 7.**
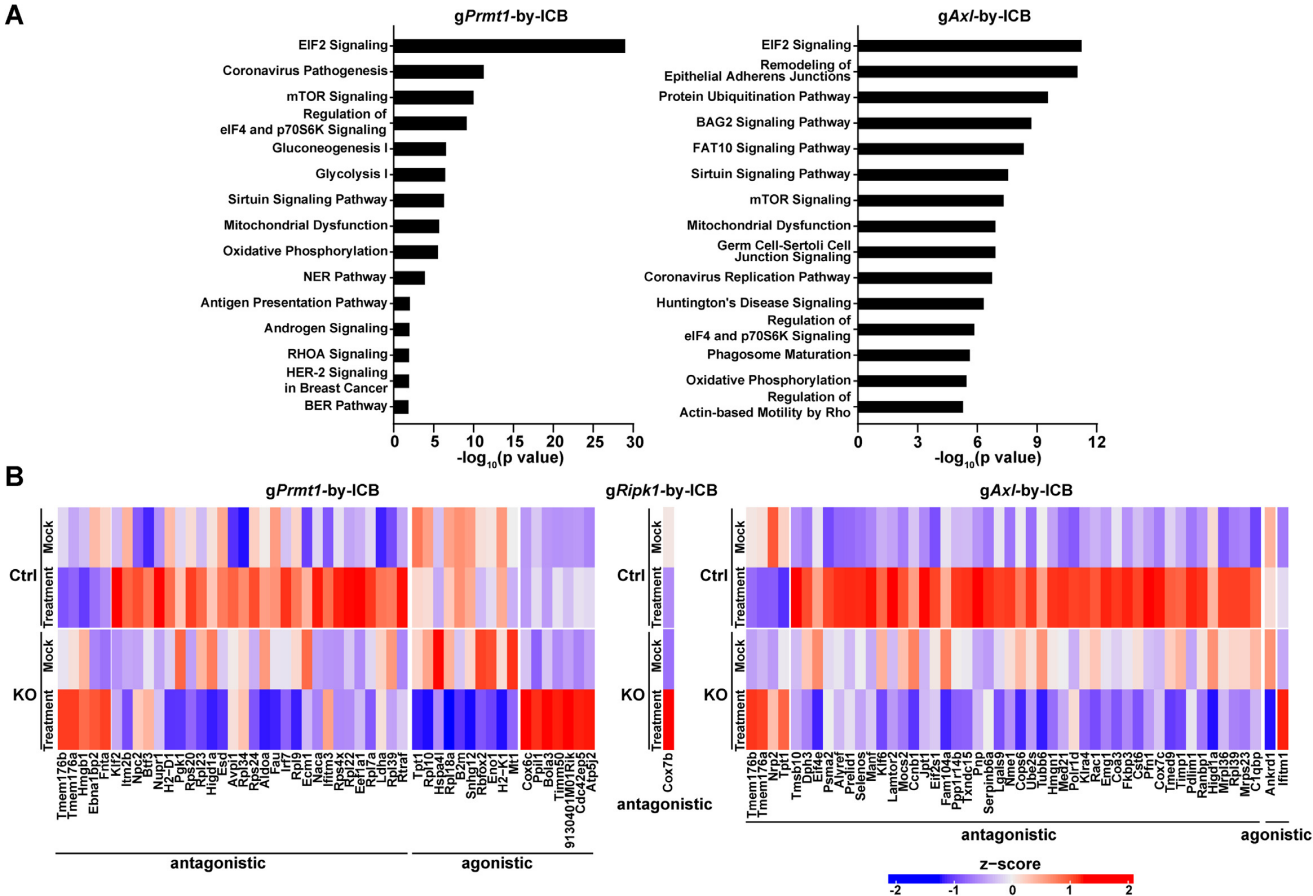
The interaction effects of gene-specific perturbations and *in vivo* anti-PD-1 treatment. (**A**) IPA of genes whose expression levels in tumor cells are modulated by KO-by-PD-1 interaction. Statistical significances of the interaction effects between genetic perturbation and *in vivo* anti-PD-1 treatment in the *in vivo*-CROP screen were determined. Genes in which the interaction effect has adjusted *P* < 0.25 were defined as ‘interaction’ genes and selected for IPA. 251 of 2179 total detected genes and 332 of 1955 total detected genes were identified as genes with interaction effect in gPRMT1 and gAxl cells, respectively. The top 15 canonical pathways displaying statistically significant interaction effects in each genetic perturbation were listed. (**B**) Heatmaps showing the relative expression levels of top ‘interaction’ genes in *Prmt1*, *Ripk1* and *Axl* KO tumor cells with or without anti-PD-1 treatment. Up to the top 50 ‘interaction’ genes with the highest fold change of the interaction effect and together with adjusted *P*-value <0.1 were listed in the heatmap.

As scCRISPR screen platforms can determine transcriptional expression at the single-cell level, a unique opportunity exists to systematically evaluate the impact of genetic perturbations on transcription in response to immune attacks. We applied both *in vitro* and *in vivo-*CROP screens to explore the interaction relationship between genetic perturbation and immune attack (Supplementary Figure S10). Similar bioinformatics approaches using interaction effect relationships have also been utilized in the identification of gene signatures of immune resistant phenotype in cancer patients (34). Genes whose expression is significantly changed by the interaction between KO and the *in vitro* T cell treatment, or the *in vivo* anti-PD-1 treatment (FDR<0.25) were defined as KO-by-T-cell genes (Supplementary Table S8) or KO-by-PD-1 genes (Supplementary Table S9), respectively. These genes were selected for ingenuity pathway analysis (Figure 6A and 7A) and over-representation analysis (ORA; Supplementary Figure S11A–D). The pathway analysis of KO-by-T-cell genes (Figure 6A) showed that genetic inhibition of these tumor intrinsic factors displays significant interaction effects on regulating several important immune pathways, such as antigen presentation and the interferon pathways (gPRMT1 and gRipk1), which was also represented from the ORA (Supplementary Figure S11A, B). Additionally, interaction effects in a couple of pathways whose immunomodulatory roles remain unclear, such as the FAT10 signaling and protein ubiquitination pathways (gPrmt1 and gRipk1) were also identified, highlighting their potential as novel molecular determinants contributing to immune resistance related to these two immune factors (Figure 6A). As we only observed significant AXL KO-by-T-cell interactions in two genes (*Pop5* and *Mcm5*), no pathway analysis was performed for gAXL cells. We also borrowed the concepts in drug development studies to categorize the types of interaction effects between genetic perturbation and immune treatment (35). The interaction effect is defined as agnostic, when genetic perturbation and immune treatment on gene expression show a similar effect, and as antagonistic, when genetic perturbation and immune treatment have a contrasting effect on the expression levels of particular genes. The changes of KO-by-T-cell genes were illustrated with either agnostic or antagonistic interaction effect of KO-by-T-cell (Figure 6B). Unlike above mentioned integrative studies (Figures 4 and 5), we observed a significant interaction effect in numerous pathways which are in common between *in vivo* anti-PD-1 treatment and *in vitro* T cell treatment, including FAT10 signaling, protein ubiquitination and oxidative phosphorylation (Figures 6A and 7A). These results suggest that knocking out these immune factors could lead to similar adaptive responses to both types of immunotherapies, whereas the mechanisms by which these factors contribute to primary resistance to immunotherapy could be variable among different forms of immunotherapy. The changes in genes with either agnostic or antagonistic interaction effect of KO-by-ICB-treatment were illustrated (Figure 7B). Overall, the list of identified pathways/genes with significant interaction effects provides a rich resource to explore the mechanisms of actions of IO combinations by targeting *PRMT1*, *RIPK1* or *AXL*.

## DISCUSSION

Immunotherapy is revolutionizing cancer treatment. However, most cancer patients still fail to respond, due in part to immunosuppressive mechanisms co-opted by tumor cells to subvert immune responses (19,36–40). To outline the landscape of tumor intrinsic factors that contribute to resistance to T-cell mediated immunity, our group and others have utilized PRISM-based pooled screens in mixtures of tumor cells expressing different types of gRNAs to functionally interrogate the immunological role of tumor intrinsic factors (4,5,10,41). By labeling each type of cancer cell with unique nucleotide barcodes, PRISM was originally designed to identify genotype-specific cancer vulnerabilities (6,42). Given that gene-specific gRNAs have unique nucleotide sequences, the application of PRISM can be easily implemented to screen phenotypes of cancer cells with genetic perturbations in response to immune attacks (4,5,10,41). However, there is a lack of systematic methods to validate candidates selected from these screens and explore their immune regulatory mechanisms. Among these candidates, only a handful of genes have confirmed their contributions to immune resistance so far.

In this study, we sought to address this throughput issue by developing scCRISPR-based immune screen platforms for target validation and mechanistic studies. Based our previous studies using modified MC38 tumors, three tumor intrinsic factors with immune regulatory roles were selected to optimize the pipelines of scCRISPR-based immune screen platforms. Based on the performance of selected factors, we evaluated the implementation of two main scCRISPR platforms, Perturb-seq and CROP-seq, in immune-related screens. Our results demonstrate that both platforms of scCRISPR immune screen can provide a high-dimensional genotypic landscape of genetic perturbations in a high-throughput manner. The transcriptional profiles of genetically modified cells defined by either scCRISPR platform well correlate with those from bulk RNA-seq. We also found that the overall best performance can be achieved when using optimized protocols for sample preparation, data preprocessing, and transformation.

Although MC38 is a carcinogen-induced colorectal cancer cell line, our previous studies (43,44) demonstrated that MC38 tumor cells display more immune-related features of human melanoma cells, such as the expression of MHC molecules and cytokine production, than available murine melanoma cell lines. The MC38-based preclinical tumor model is commonly used to address immune-related questions in melanomas. Therefore, results from melanoma patients receiving either ACT or ICB were used for integrative analysis. Our integrative studies and analyses of interaction effects also revealed the power of scCRISPR immune screens in discovering prognostic biomarkers for cancer immunotherapy and identifying novel tumor immune resistance mechanisms. Moreover, our unpublished data showed that scCRISPR-compatible vectors selected in this study can efficiently induce gene inactivation in T cells. We expect that scCRISPR immune screens can also be applied to evaluate the direct impact of these targets on immune cell function, which is important for the rational design of IO combinations.

However, we did not observe significant depletion of *Prmt1*, *Ripk1* or *Axl* KO cells in pooled samples after being treated with T cells by using scRNA-seq, whereas results from genomic barcode sequencing used in conventional PRISM-based pooled screens show that *Prmt1*-KO cells are consistently more sensitive to T cell killing. This suggests that scCRISPR immune screen platforms are less sensitive to phenotypic changes than conventional CRISPR immune screen platforms if the abundance of gRNA-expressing cells is used as a solo readout. Limited cell number for sequencing and potential bias introduced by cell sorting in scCRISPR platforms might contribute to a reduced capability of scCRISPR platforms to characterize phenotypic changes. Therefore, it is better to integrate genomic barcode sequencing in scCRISPR immune screens to appropriately reflect the distribution of gRNA-expressing cells in pooled samples. Moreover, when we generated tumor cell lines expressing gRNAs for scCRISPR immune screens in this study, viral transduction efficiency was not monitored. Unlike our conventional PRISM-based CRISPR screens (10), tumor cells used for scCRISPR immune screens might contain more than one copy of gRNA sequence, which might confound gRNA distribution in pooled samples. This possibility is supported by the fact that results from genomic barcoding sequencing failed to reproduce the altered vulnerability of gRipk1 and gAxl cells to T cell killing as shown in our previous studies (10,19). A well-controlled viral transduction procedure is likely required to further improve the performance of scCRISPR immune screens. Additionally, although gRNA-expressing cells were separately generated, we still observed some of cells with multiple types of gRNA reads in all our screens. It suggests that the procedure of single cell partitioning cannot eliminate multiplets or cells contaminated with RNAs from other cells.

Although the change of abundance of each gRNA-expressing cell line in pooled samples is not a reliable readout in scCRISPR immune screens, we found that the distribution pattern of gPrmt1 cells in clusters assigned by transcriptional profiles is significantly different as that of gNC cells. More dramatic changes of cluster distribution in gPrmt1 cells in response to immune treatment were observed. Furthermore, when we compared the impacts of *Prmt1*, *Ripk1* or *Axl* inhibition on the expression of DEGs in pre-treated tumor samples between responders and non-responders to ACT and ICB, our results showed that perturbing these factors tends to modify tumor transcriptional profiles towards the profiles favoring antitumor immune responses. These results suggest that the transcriptional profiles revealed in scCRISPR immune screens can be used to outline the phenotypic landscape of targeting tumor intrinsic factors in response to cancer immunotherapy and predict therapeutic potentials of new IO combinations. More importantly, our analysis of interaction effects not only showed that inhibiting *Prmt1* or *Ripk1* can modulate activities of antigen presentation and interferon signaling pathways, which were further validated by recently published studies (10,44,45), but also found a series of pathways and genes whose immunological function remains unclear. Given that the interaction effects of genetic perturbation and immunotherapy cannot be easily evaluated by conventional molecular methods, scCRISPR immune screens have a great potential to identify novel tumor immune evasion mechanisms.

Finally, our recommendations for the experimental design of scCRISPR screens were summarized in the Supplementary Figure S12. For the Perturb-seq, results from our group and others clearly showed that the capture efficiency of gRNA transcripts greatly varies related to the capture sequence selection and its relative location. Therefore, the optimization of gRNA scaffold design is strongly recommended for users who would like to choose the Perturb-seq platform. On the other hand, the CROP-seq is smartly designed to avoid the need for adding capture sequences. By leveraging long terminal repeat (LTR)-mediated integration, lentivirus-transduced cells can express three types of transcripts with gRNA sequences including (i) the original copy of hU6-driven gRNA (no polyA tail), (ii) a genome integration copy of hU6-driven gRNA (no polyA tail) and (iii) an EF1a-driven long mRNA with gRNA sequence (with polyA tail). Given that the 3′ single-cell sequencing protocol can only capture RNAs with polyA tails, the CROP-seq uses the EF1a-driven long mRNAs for gRNA assignment. Our results showed that the expression level of the EF1a-driven long mRNAs is significantly higher than the expression of gRNAs driven by the U6 promoter, particularly under the transduction condition using the low viral titer. Thus, higher expression level of gRNA transcript from the CROP-seq platform can be achieved that that from the Perturb-seq platform. Therefore, the CROP-seq is more suitable for large-scale pooled gRNA screens in which low viral titer is required for transduction. Furthermore, when we directly transfected the CROP-seq vector into 293T cells with Cas9 expression, gene-specific knockout cannot be observed in transfected cells (data not shown). It suggests that the original copy of hU6-driven gRNA might not be sufficient to achieve a successful gene editing.

scCRISPR immune screen platforms can be further improved from the following aspects, including capture rates of gRNA transcripts, reads on mRNAs with low abundance, and reads on non-coding RNAs. Recently, a 5′ direct capture Perturb-seq by paring 5′ end sequencing technology with the Perturb-seq platform has been developed and commercialized by 10× Genomics (18). In a recent study (14), 3′-direct and 5′-direct Perturb-seq methods were directly compared in parallel. Their results showed that the gRNA capture efficiency of different scaffold designs in the 5′-direct Perturb-seq platform are comparable and higher than those in the 3′-direct Perturb-seq platform. Furthermore, TCR/BCR repertoire analysis can only be achieved by 5′-direct scRNA-seq. Therefore, 5′-direct Perturb-seq immune screens have the potential to determine impacts of genetic perturbation on T/B-cell clonality. In addition, this new platform might also expand the coverage of transcriptional profiles, which will be evaluated in future studies.

Taken together, in this proof-of-principle study, we optimized scCRISPR immune screen platforms and confirmed their utility in better understanding tumor immune evasion mechanisms and developing novel IO combinations. scCRISPR immune screen platforms are capable to offer novel insights regarding the underlying mechanisms by which tumor intrinsic factors contribute to cancer immune resistance.

## DATA AVAILABILITY

Raw read counts from sequencing data are accessible from GEO (GSE206107).

## Supplementary Material

zcac038_Supplemental_Files
