# Supplementary material for: Single-cell CRISPR immune screens reveal immunological roles of tumor intrinsic factors
Source: NAR Cancer. 2022 Dec 9;4(4):zcac038. doi: 10.1093/narcan/zcac038 (PMC9732527; doi:10.1093/narcan/zcac038)
Supplement: zcac038_Supplemental_Files [file zcac038_supplemental_files.zip › 2022-10-15 supplementary figures+legends.pdf]

	Perturb-Seq	CROP-Seq
Vector Design	Generate the vector with the specific capture sequence at certain location	Using the designated vector
gRNA Capture	gRNAs without polyA tails transcribed by Pol III	gRNAs with polyA tails transcribed by Pol II
Compatible Kit	10x Chromium Single Cell 3' Feature Barcode Library Kit	10x Chromium Next GEM Single Cell 3' Kit

Procedures of Library Construction

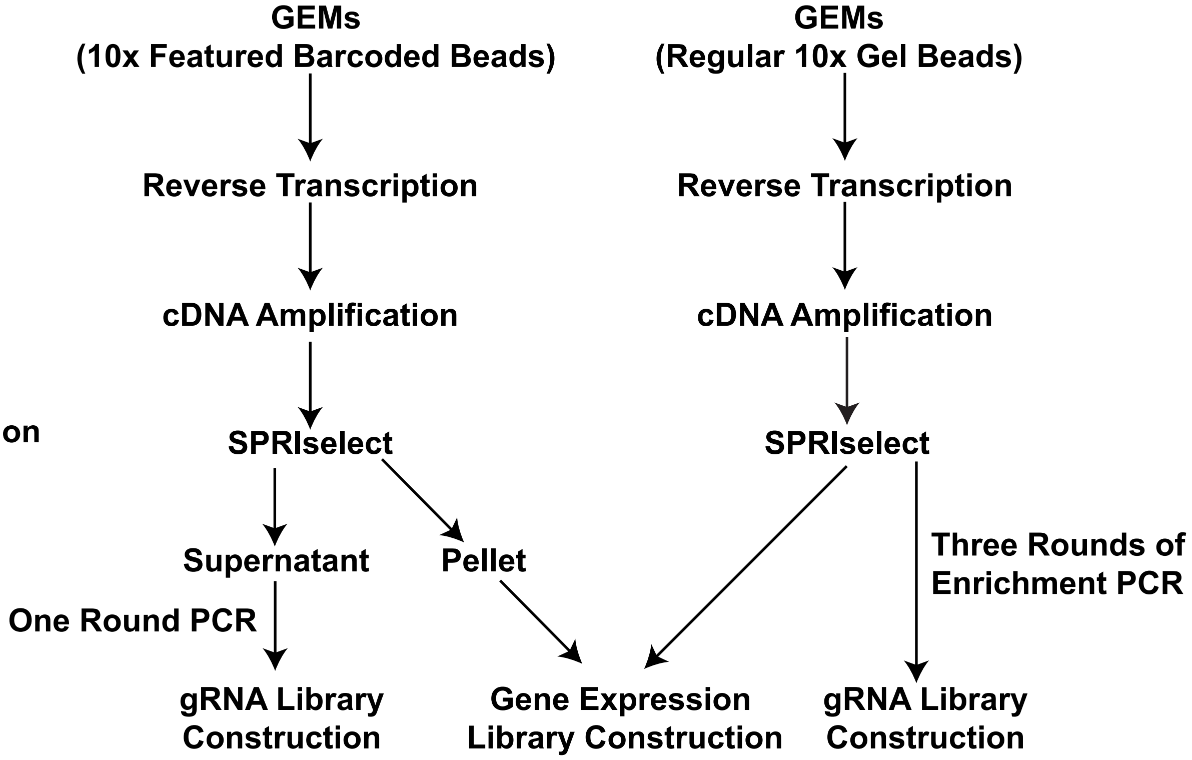

JHou et.al Supplementary Figure 2

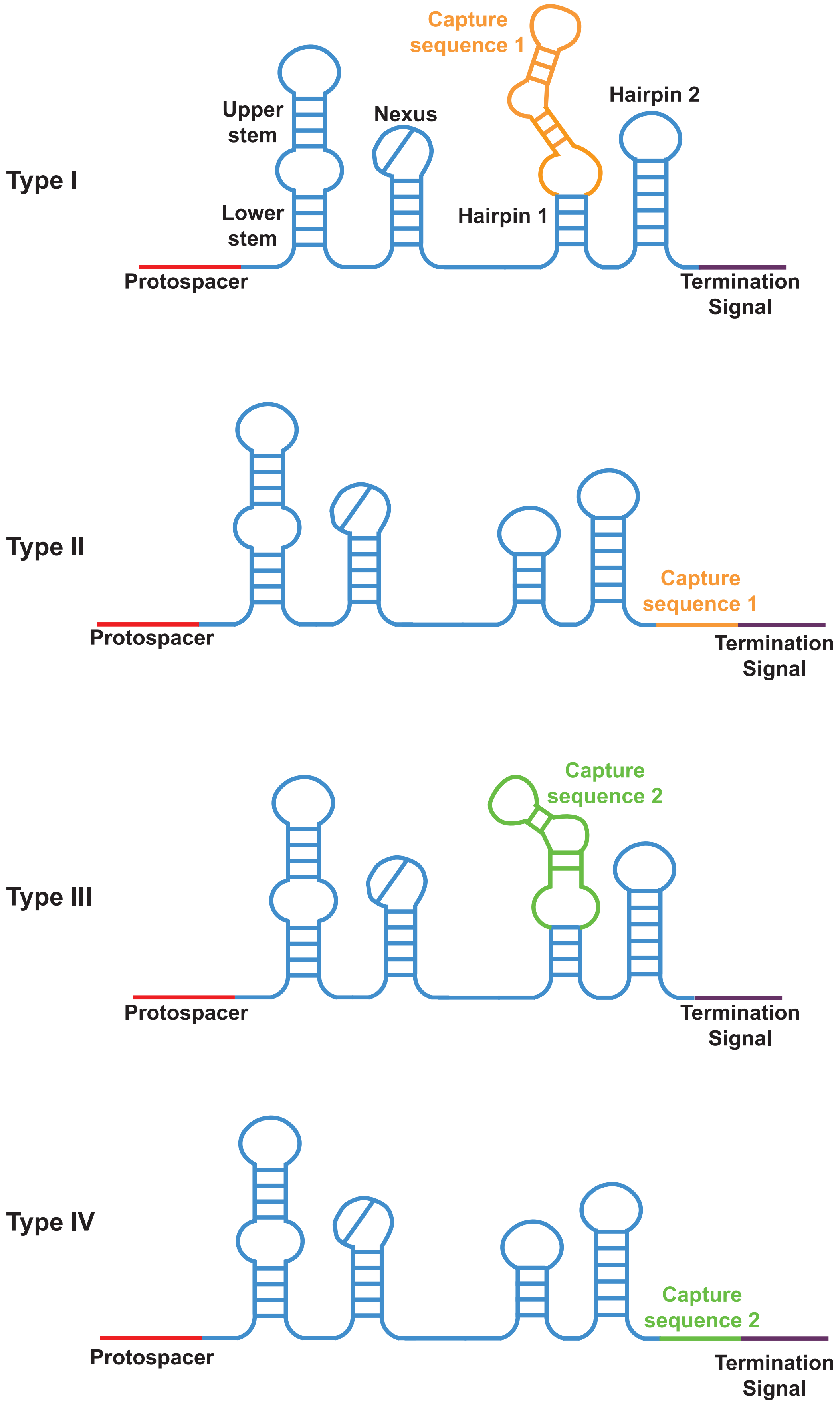

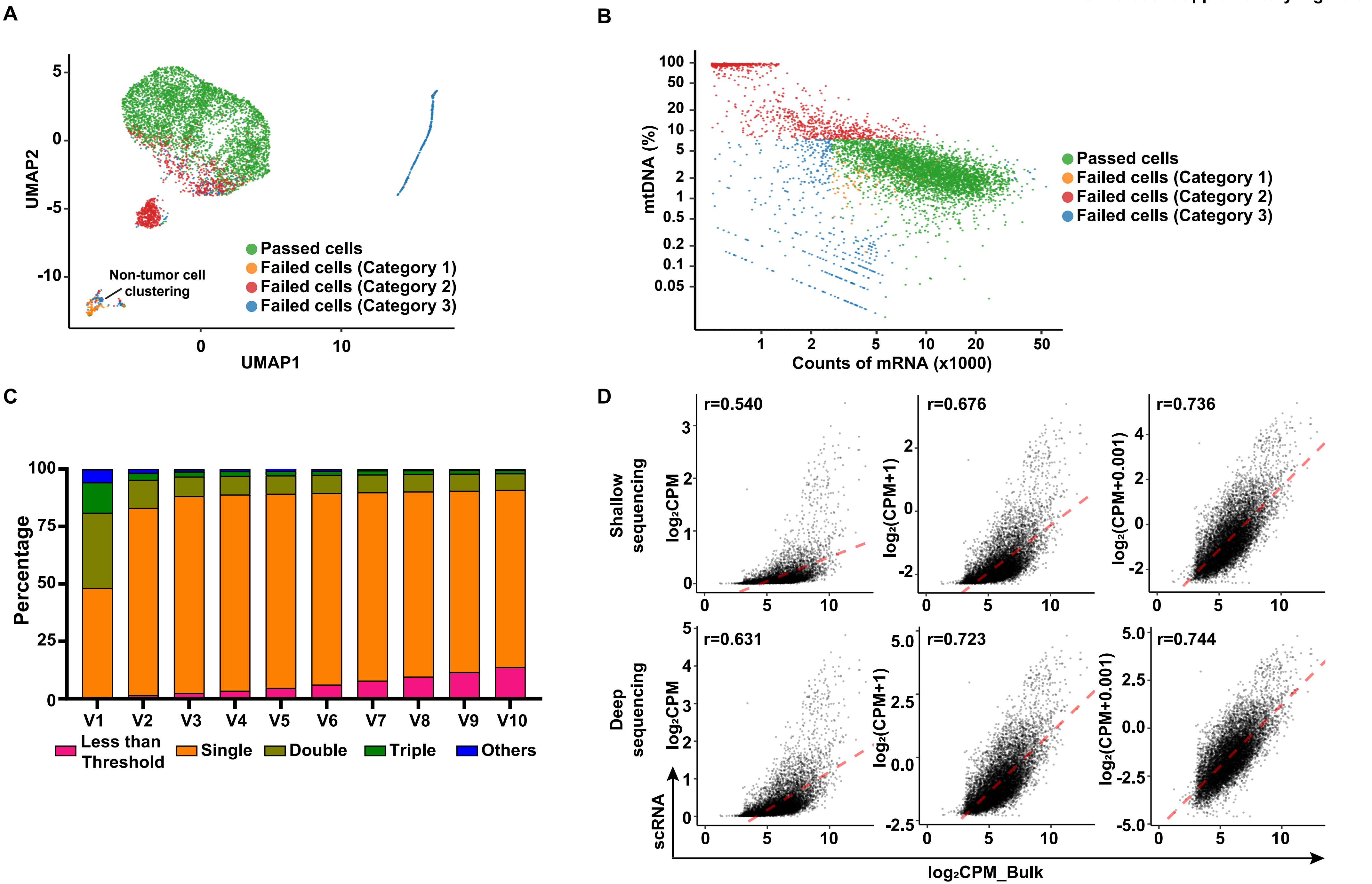
A
UMAP2
UMAP1
Non-tumor cell clustering
Passed cells
Failed cells (Category 1)
Failed cells (Category 2)
Failed cells (Category 3)
B
mtDNA (%)
Counts of mRNA (x1000)
Passed cells
Failed cells (Category 1)
Failed cells (Category 2)
Failed cells (Category 3)
C
Percentage
V1 V2 V3 V4 V5 V6 V7 V8 V9 V10
Less than Threshold
Single
Double
Triple
Others
D
Shallow sequencing
Deep sequencing
r=0.540
r=0.676
r=0.736
r=0.631
r=0.723
r=0.744
log₂CPM
log₂(CPM+1)
log₂(CPM+0.001)
scRNA
log₂CPM_Bulk

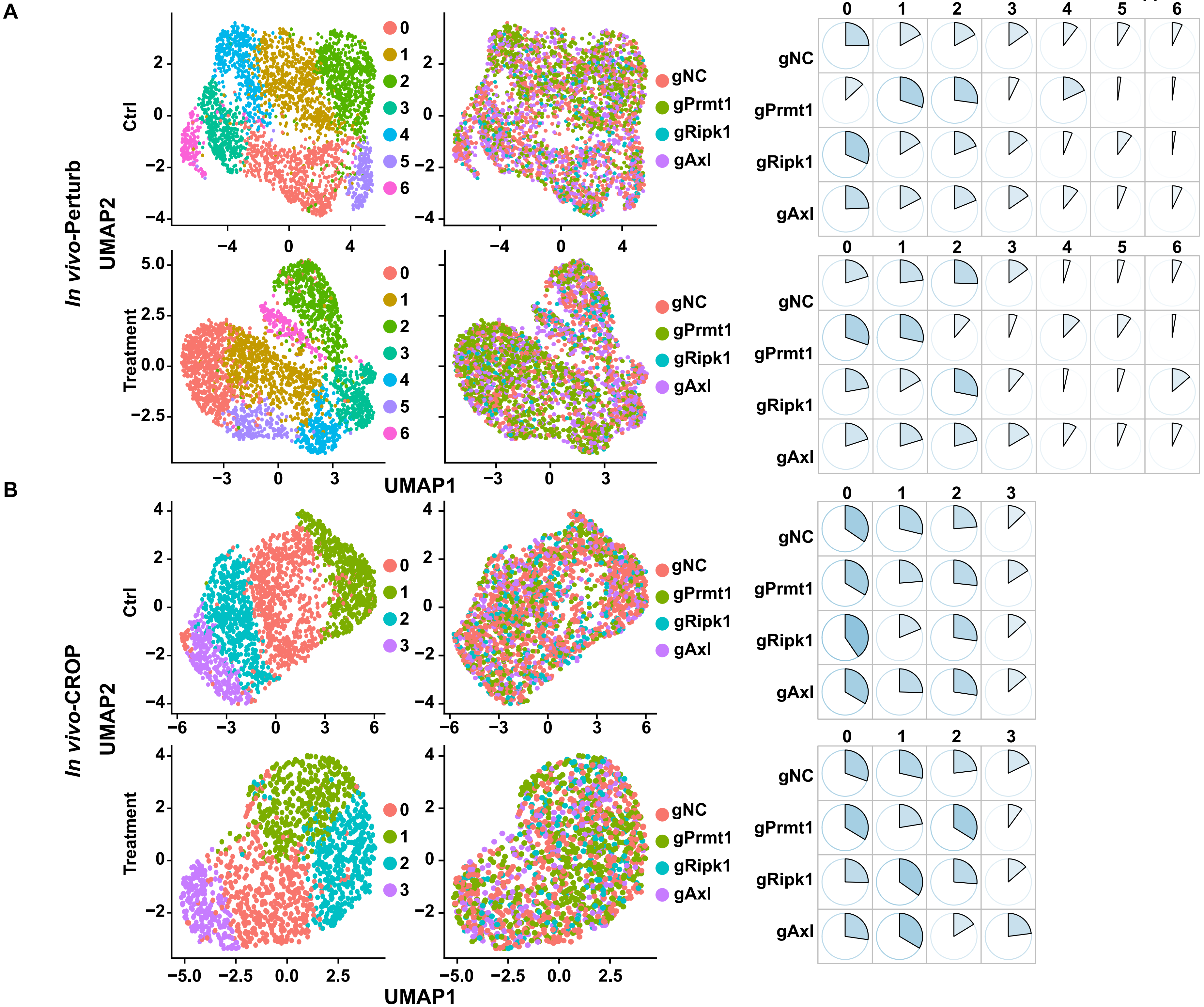
A
B
In vivo-Perturb
In vivo-CROP
UMAP2
UMAP1
Ctrl
Treatment
0
1
2
3
4
5
6
gNC
gPrmt1
gRipk1
gAxl

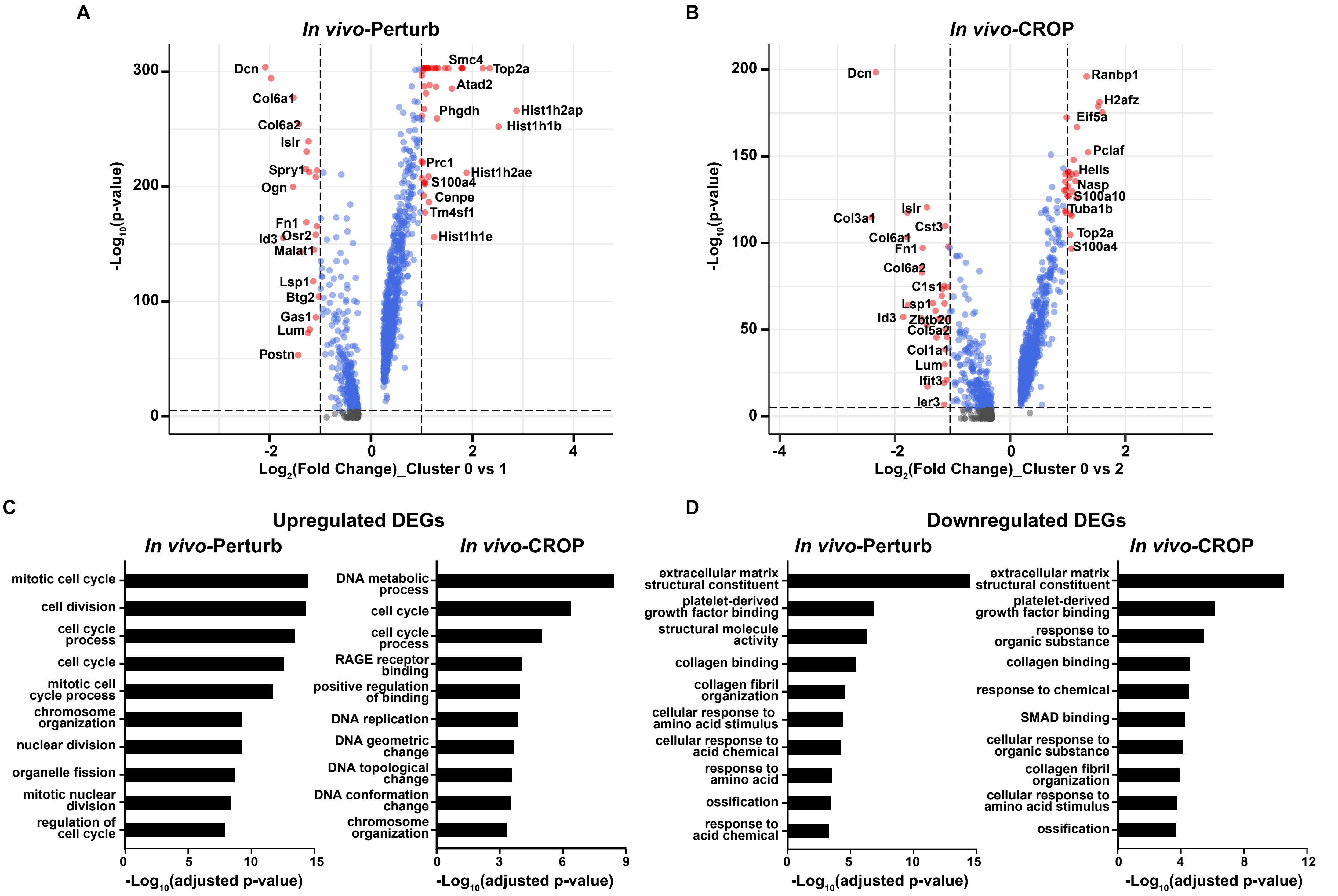
A
In vivo-Perturb
-Log10(p-value)
Log2(Fold Change)_Cluster 0 vs 1
Dcn
Col6a1
Col6a2
Islr
Spry1
Ogn
Fn1
Id3
Osr2
Malat1
Lsp1
Btg2
Gas1
Lum
Postn
Smc4
Top2a
Atad2
Phgdh
Hist1h2ap
Hist1h1b
Prc1
Hist1h2ae
S100a4
Cenpe
Tm4sf1
Hist1h1e
B
In vivo-CROP
-Log10(p-value)
Log2(Fold Change)_Cluster 0 vs 2
Dcn
Islr
Col3a1
Cst3
Col6a1
Fn1
Col6a2
C1s1
Lsp1
Id3
Zbtb20
Col5a2
Col1a1
Lum
Ifit3
Ier3
Ranbp1
H2afz
Eif5a
Pclaf
Hells
Nasp
S100a10
Tuba1b
Top2a
S100a4
C
Upregulated DEGs
In vivo-Perturb
mitotic cell cycle
cell division
cell cycle process
cell cycle
mitotic cell cycle process
chromosome organization
nuclear division
organelle fission
mitotic nuclear division
regulation of cell cycle
-Log10(adjusted p-value)
In vivo-CROP
DNA metabolic process
cell cycle
cell cycle process
RAGE receptor binding
positive regulation of binding
DNA replication
DNA geometric change
DNA topological change
DNA conformation change
chromosome organization
-Log10(adjusted p-value)
D
Downregulated DEGs
In vivo-Perturb
extracellular matrix structural constituent
platelet-derived growth factor binding
structural molecule activity
collagen binding
collagen fibril organization
cellular response to amino acid stimulus
cellular response to acid chemical
response to amino acid
ossification
response to acid chemical
-Log10(adjusted p-value)
In vivo-CROP
extracellular matrix structural constituent
platelet-derived growth factor binding
response to organic substance
collagen binding
response to chemical
SMAD binding
cellular response to organic substance
collagen fibril organization
cellular response to amino acid stimulus
ossification
-Log10(adjusted p-value)

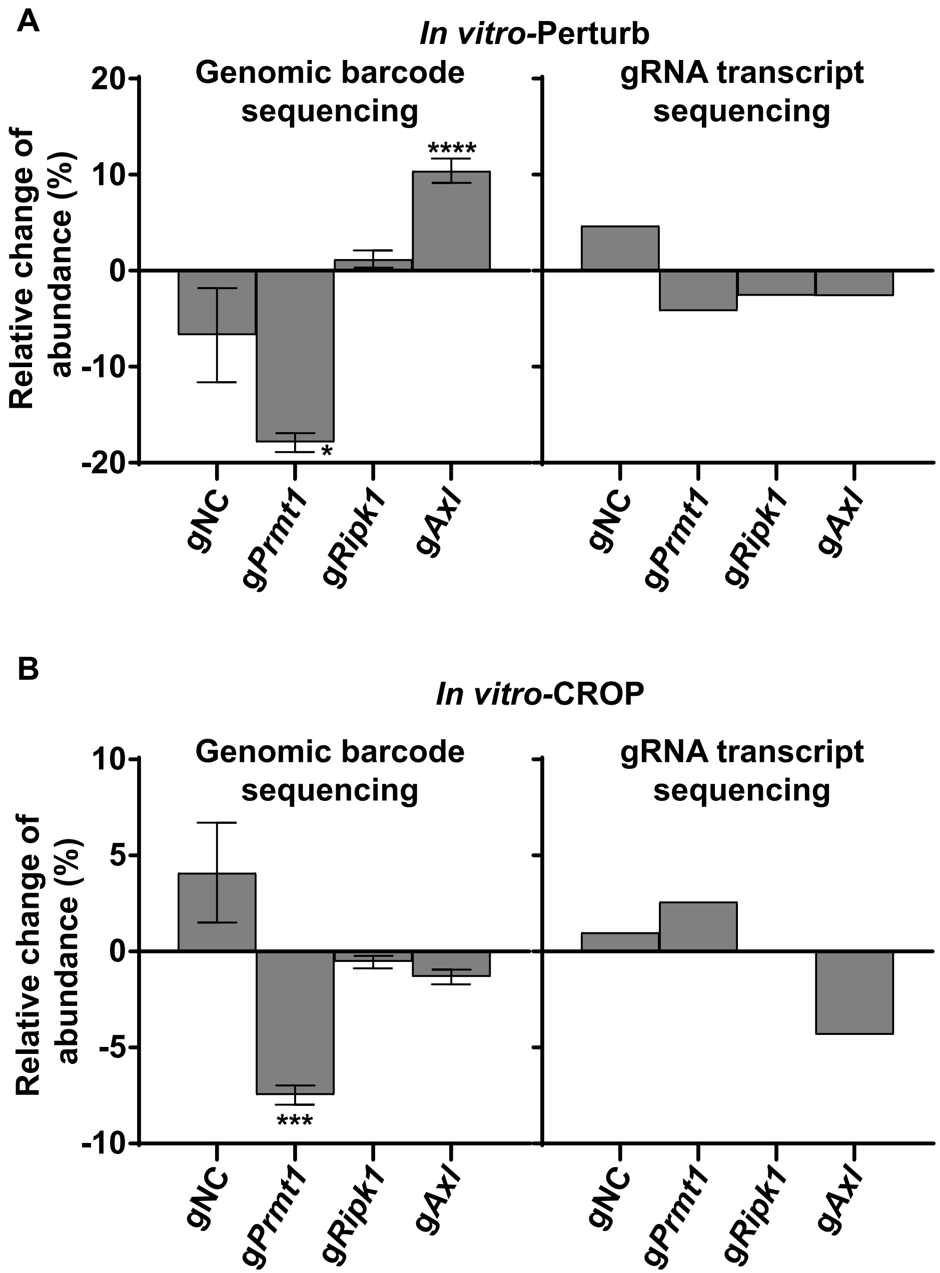
A
In vitro-Perturb
Genomic barcode sequencing
gRNA transcript sequencing
Relative change of abundance (%)
20
10
0
-10
-20
****
*
gNC
gPrmt1
gRipk1
gAxl
B
In vitro-CROP
Genomic barcode sequencing
gRNA transcript sequencing
Relative change of abundance (%)
10
5
0
-5
-10
***
gNC
gPrmt1
gRipk1
gAxl

A

Control

	Accuracy	p-value
gNC1 vs gNC2	0.51	0.21
gNC vs gPrmt1	0.71	0.027
gNC vs gRipk1	0.65	0.338
gNC vs gAxl	0.66	0.201

Treatment

	Accuracy	p-value
gNC1 vs gNC2	0.51	0.286
gNC vs gPrmt1	0.73	<0.001
gNC vs gRipk1	0.72	<0.001
gNC vs gAxl	0.66	0.398

B

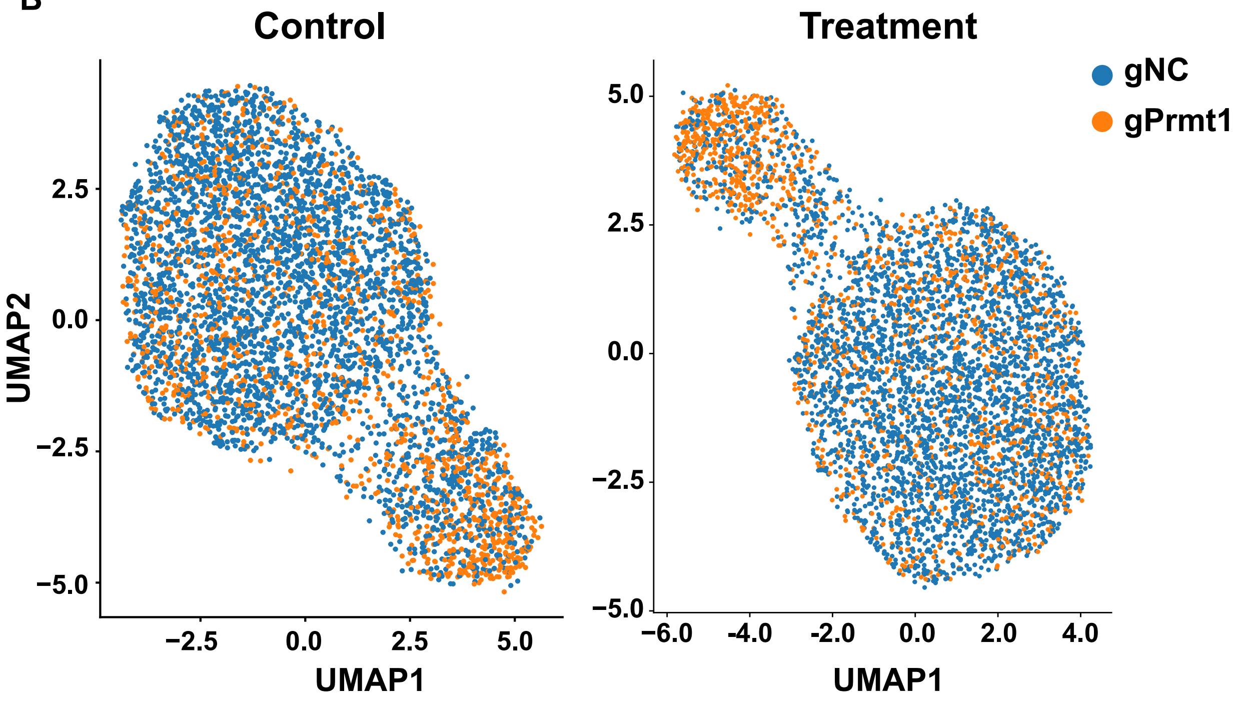

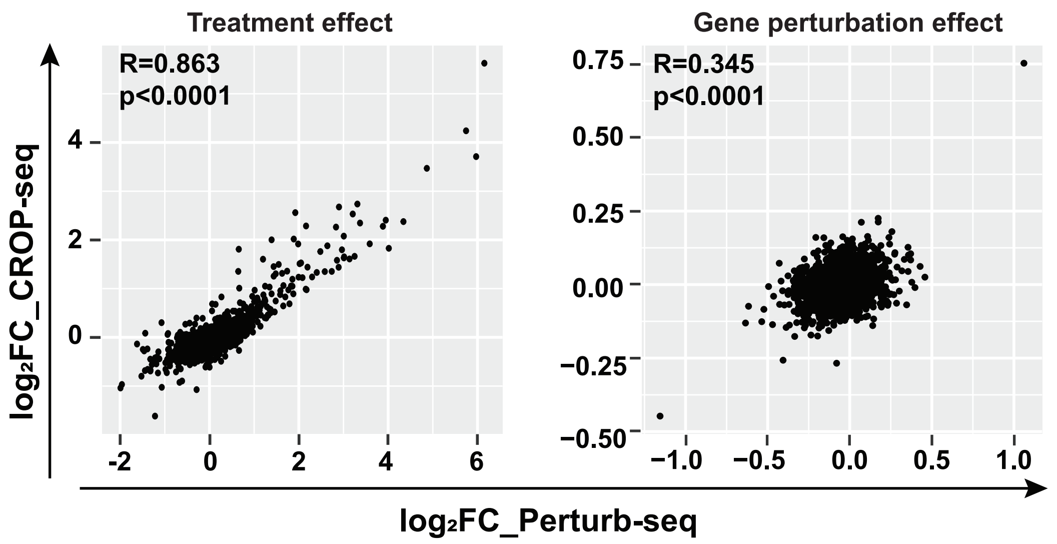
Treatment effect
Gene perturbation effect
R=0.863
p<0.0001
R=0.345
p<0.0001
4
2
0
-2
0
2
4
6
0.75
0.50
0.25
0.00
−0.25
−0.50
−1.0
−0.5
0.0
0.5
1.0
log₂FC_CROP-seq
log₂FC_Perturb-seq

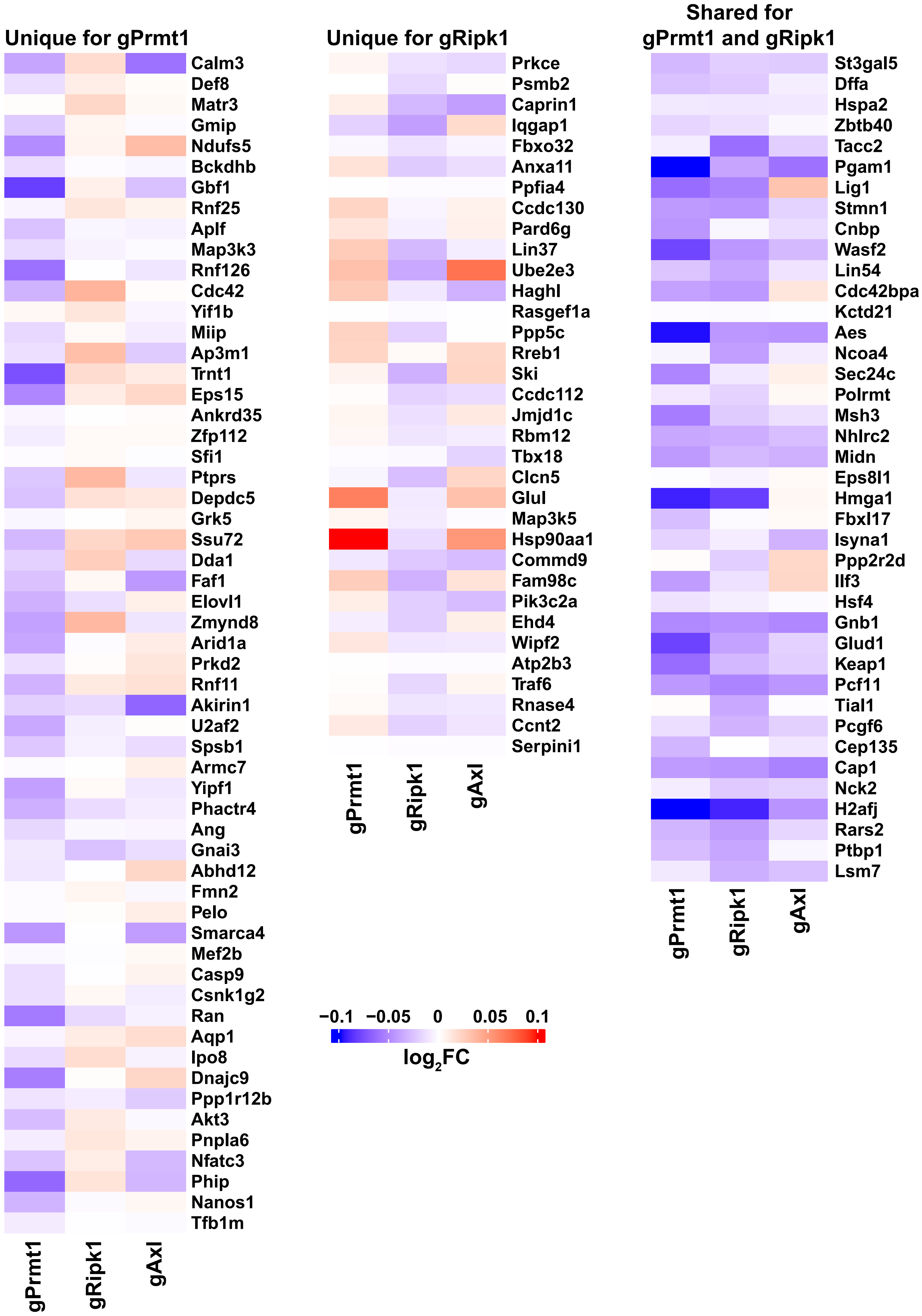
Unique for gPrmt1
Calm3
Def8
Matr3
Gmip
Ndufs5
Bckdhb
Gbf1
Rnf25
Aplf
Map3k3
Rnf126
Cdc42
Yif1b
Miip
Ap3m1
Trnt1
Eps15
Ankrd35
Zfp112
Sfi1
Ptprs
Depdc5
Grk5
Ssu72
Dda1
Faf1
Elovl1
Zmynd8
Arid1a
Prkd2
Rnf11
Akirin1
U2af2
Spsb1
Armc7
Yipf1
Phactr4
Ang
Gnai3
Abhd12
Fmn2
Pelo
Smarca4
Mef2b
Casp9
Csnk1g2
Ran
Aqp1
Ipo8
Dnajc9
Ppp1r12b
Akt3
Pnpla6
Nfatc3
Phip
Nanos1
Tfb1m
gPrmt1
gRipk1
gAxl
Unique for gRipk1
Prkce
Psmb2
Caprin1
Iqgap1
Fbxo32
Anxa11
Ppfia4
Ccdc130
Pard6g
Lin37
Ube2e3
Haghl
Rasgef1a
Ppp5c
Rreb1
Ski
Ccdc112
Jmjd1c
Rbm12
Tbx18
Clcn5
Glul
Map3k5
Hsp90aa1
Commd9
Fam98c
Pik3c2a
Ehd4
Wipf2
Atp2b3
Traf6
Rnase4
Ccnt2
Serpini1
gPrmt1
gRipk1
gAxl
−0.1 −0.05 0 0.05 0.1
log2FC
Shared for gPrmt1 and gRipk1
St3gal5
Dffa
Hspa2
Zbtb40
Tacc2
Pgam1
Lig1
Stmn1
Cnbp
Wasf2
Lin54
Cdc42bpa
Kctd21
Aes
Ncoa4
Sec24c
Polrmt
Msh3
Nhlrc2
Midn
Eps8l1
Hmga1
Fbxl17
Isyna1
Ppp2r2d
Ilf3
Hsf4
Gnb1
Glud1
Keap1
Pcf11
Tial1
Pcgf6
Cep135
Cap1
Nck2
H2afj
Rars2
Ptbp1
Lsm7
gPrmt1
gRipk1
gAxl

Total= CRISPR + Treatment + Interaction

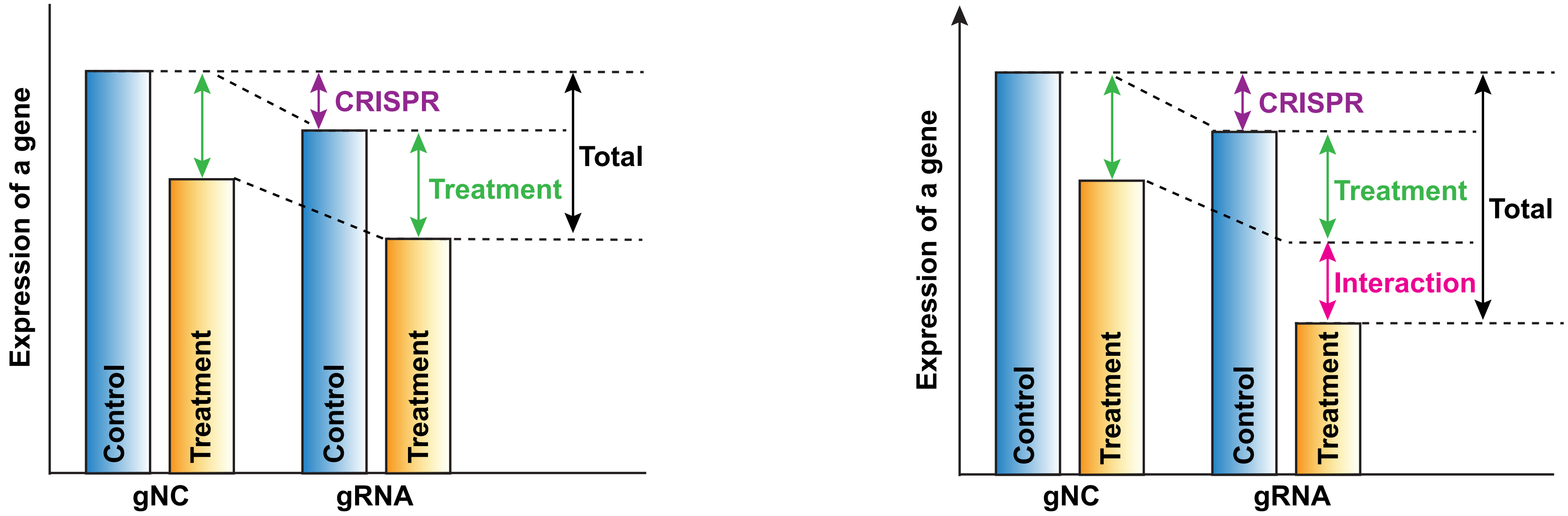

Interaction is not present
Treament has the same effect on both gNC and gRNA cells

Interaction is not present
Treament has an additional effect on gRNA cells

JHou et.al Supplementary Figure 11

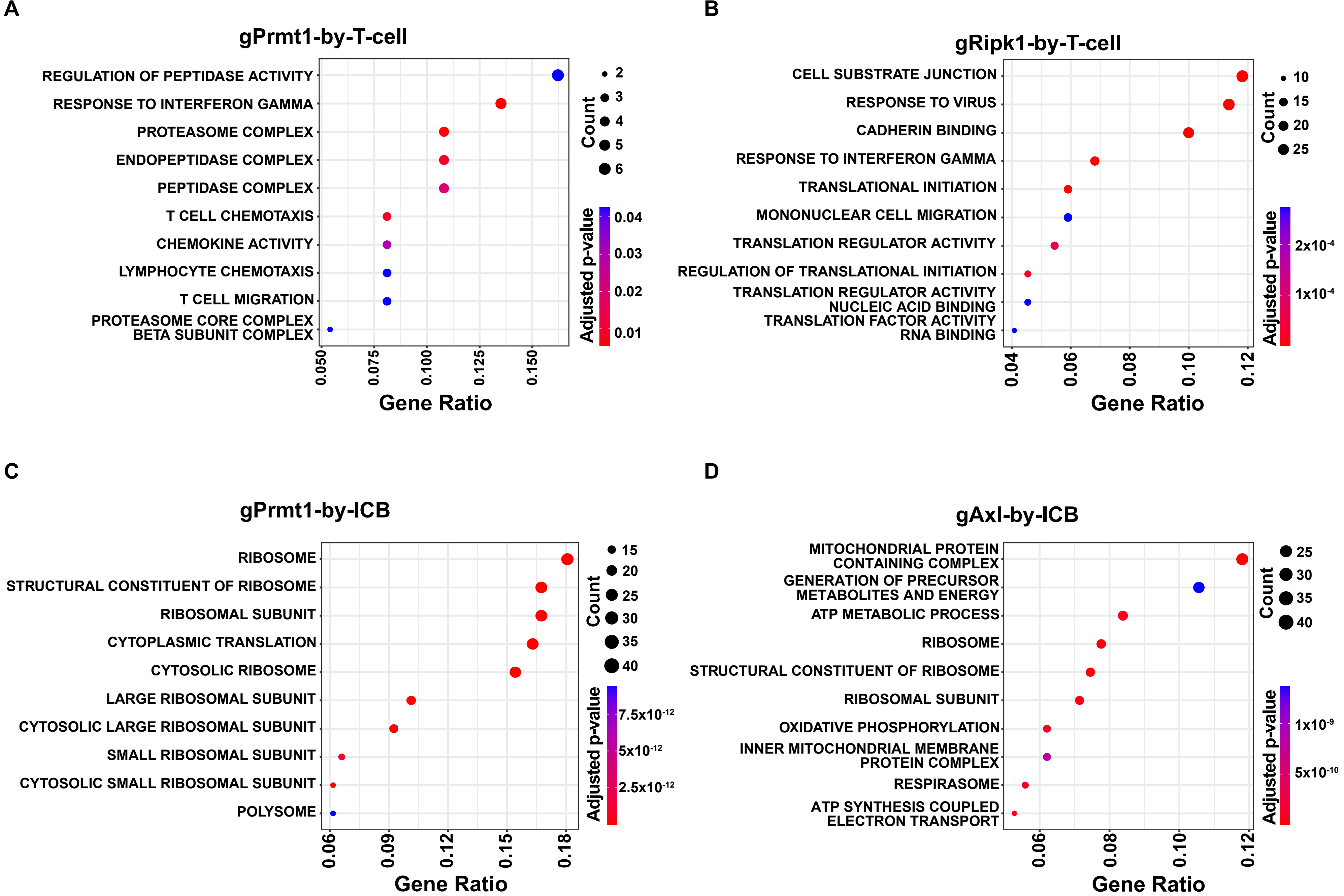

<table>
<tr><th></th><th>Perturb-Seq</th><th>CROP-Seq</th></tr>
<tr><td>Vector Optimization</td><td>Required</td><td>Not required</td></tr>
<tr><td>Virus preparation for gRNA transduction</td><td>Relative higher MOI required</td><td>No requirement</td></tr>
<tr><td>Cell preparation</td><td colspan="2">Using FACS to exclude dead cells and non-targeted cells before single cell isolation</td></tr>
<tr><td>Cell viability</td><td colspan="2">>85%</td></tr>
<tr><td>Sequenced Cell number</td><td colspan="2">>100 per gRNA</td></tr>
<tr><td>GEX library preparation</td><td>10x Chromium Single Cell 3' Feature Barcode Library Kit</td><td>10x Chromium Next GEM Single Cell 3' Kit</td></tr>
<tr><td>gRNA library preparation</td><td>10x Chromium Single Cell 3' Feature Barcode Library Kit</td><td>GEX library
+
Three rounds of customized PCR for gRNA enrichment</td></tr>
<tr><td rowspan="2">Sequencing Requirement</td><td colspan="2">> 15,000 raw read pairs per cell to GEX library</td></tr>
<tr><td colspan="2">> 5,000 raw read pairs per cell to gRNA library</td></tr>
<tr><td>Data filtering</td><td colspan="2">Perform the bioinformatics pipeline with
• Exclude non-targeted cells
• Exclude dead cells by using the cut-off of mtDNA >7.5%
• Exclude low-quality cells and multiplets by using the cut-off of mRNA counts of total sequenced cells <5% and >95%
• Select high-quality cells (gRNA number ≥3 per cell, type=1)
• Transform gene expression data (CPM) into $\log_2$(CPM+0.001)</td></tr>
</table>

Supplementary Figure 1. Comparisons between the Perturb-seq platform and the CROP-seq platform. GEMs stands for Gel Beads-In-Emulsions and SPRI stands for Solid Phase Reversible Immobilization.

Supplementary Figure 2. Schematic diagrams showing four types of gRNA scaffold designs for gRNA-expressing vectors that are compatible with Perturb-seq.

Supplementary Figure 3. Optimization of bioinformatics analysis pipeline for scCRISPR immune screens. (A) A representative UMAP plot of the *in vivo*-CROP screen shows cell clusters before data filtering. A set of filters were applied to select tumor cells with high-quality sequencing data for further process (Passed cells). Cells expressing the gene signature of immune cells (category 1), cells with a percentage of mtDNA larger than 7.5(category 2), and cells whose mRNA counts are either lower than 5% or more than 95% of total sequenced cells (category 3) were defined as failed cells. (B) A representative plot showing a negative association between the mtDNA percentage and mRNA count in individual cells. Data for representation were obtained from the *in vivo*-CROP screen. (C) The yield and accuracy of gRNA assignments under different thresholds of gRNA counts. The threshold of gRNA counts per cell for gRNA assignments is set at a range from 1 to 10 (V1-V10). Under different thresholds, the percentages of cells with one type of gRNAs (single) and cells with more than one type of gRNAs (double, triple, and others) were listed. (D) Correlations between scRNA-seq results and bulk RNA-seq results. Results from *in vitro*-CROP screens under different sequence conditions were used to optimize constants for $\log_2$-transformation. The constants selected for the test are 0, 1, and 0.001. Gene expression levels were determined by $\log_2$ (Count + c) and $\log_2$ (Count) in scRNA-seq results and bulk RNA-seq results, respectively. scRNA-seq results were obtained from *in vitro*-CROP screens.

Supplementary Figure 4: The distributions of gRNA-expressing tumor cells in the control group and the treatment group from the *in vivo*-Perturb screen (A) and the *in vivo*-CROP screen (B).

Supplementary Figure 5. Differentially expressed genes (DEGs) between treatment-enriched clusters and control-enriched clusters in both screen platforms. (A-B) Volcano plots showed identified DEGs between treatment-enriched clusters and control-enriched clusters from the *in vivo*-Perturb screen (A) and the *in vivo*-CROP screen (B). The Log_2 fold change and Log_{10} P-value from Wilcoxon test of each gene expression between treatment-enriched clusters and control-enriched clusters were calculated. Dashed line of the Y-axis indicated p-value=0.05; Dashed lines of the X-axis indicated $|Log_2 \text{(Fold change)}|=1$. Genes with p-value>0.05 were labeled with grey dots. Genes with p-value<0.05 and $|Log_2 \text{(Fold change)}|>1$ were identified as DEGs and labeled with red dots. The remaining genes were labeled with blue dots. (C-D) Gene ontology analysis of upregulated gene (C) and downregulated genes (D) in treatment-enriched clusters from *in vivo* scCRISPR immune screens. The top 10 GO profiles were illustrated based on the adjusted p-value for illustration.

Supplementary Figure 6. Effects of genetic perturbation on tumor sensitivity to T cell killing. The percentages of genomic gRNA reads and gRNA-expressing cells in pooled tumor samples were determined by genomic barcode sequencing and scRNA-seq, respectively. The effect of each genetic perturbation on tumor sensitivity to T cell killing was quantified by the relative change of gRNA abundance between samples with and without T cell treatment. Relative change of abundance= (gRNA% in treated samples- gRNA% in control samples)/ gRNA% in control samples. Data for representation were obtained from the *in vitro*-Perturb screen (A) and the *in vitro*-CROP screen (B). *p<0.05; *** p<0.001, and ****p<0.0001.

Supplementary Figure 7. Cell clustering analysis based on gRNA identity. (A) The accuracy scores and related p-values based on a random forest classifier training model to predict each perturbation were listed. (B) A supervised UMAP plot of the distribution of gNC-expressing cells and gPrmt1-expressing cells obtained from the *in vitro*-CROP screen.

Supplementary Figure 8. Consistent transcriptional profiles were obtained from Perturb-seq and CROP-seq screens. Results from the *in vitro*-Perturb screen and the *in vitro*-CROP screen were used to determine a correlation between the changes of transcriptional profiles defined by Perturb-seq and those by CROP-seq in response to treatment or genetic perturbation. For treatment effects, the dot plot was graphed using means of log2 fold changes of gene expression in each type of gRNA-expressing cells between the control group and the treatment group. Whereas means of log2 fold changes in gene expression between gRNA cells and gNC cells were used to plot for illustrating genetic perturbation effects.

Supplementary Figure 9. Expression levels of identified genes associated ACT response by different genetic perturbations. Genes associated ACT response in melanoma patients were selected and stratified into three categories, “Unique for gPrmt1”, “Unique for gRipk1” and “Shared for gPrmt1 and gRipk1”, as described in Figure 4. Heatmaps illustrated the $\log_2$fold changes (FC) of gene expression of each selected gene in gPrmt1, gRipk1 and gAxl cell lines when compared with gNC.

Supplementary Figure 10. Schematic illustration of interaction relationships of CRISPR perturbation and treatment. The left panel illustrates a scenario where no interaction is detected. The effect of CRISPR perturbation (purple) and treatment (green) are simply additive. The right panel illustrates a scenario where interaction is present. When both CRISPR (purple) and treatment

(green) are applied, in addition to the sum of their effects, an additional interaction effect (pink) is present.

Supplementary Figure 11: Over-representation analysis (ORA) of genes whose expression levels in tumor cells were modulated by KO-by-Treatment interaction. (A-B) ORA analysis of genes whose expression levels in tumor cells are modulated by KO-by-T cell interaction. (C-D) ORA analysis of genes whose expression levels in tumor cells are modulated by KO-by-ICB interaction. The top 10 GO profiles from C5 category including three major sub-ontologies of GO_Molecular Function, GO_Cellular Component, and GO_Biological Process from each genetic perturbation were illustrated.

Supplementary Figure 12: List of recommended conditions for scCRISPR immune screens.

Supplementary Table 1. List of sequences of synthetic DNA fragments to construct gRNA-expressing vectors for Perturb-seq. To optimize gRNA scaffold designs in gRNA-expressing vectors for Perturb-seq, four types of backbone vectors were designed and constructed**.** Sequences of synthetic DNA fragments (5'-3') used for vector construction were listed.

Supplementary Table 2. List of DNA sequences used to construct gene-specific gRNAs. Both protospacer sequence and flanking sequences used to construct gRNAs targeting *Prmt1*, *Ripk1*, *Axl*, and two non-targeting gRNAs were listed.

Supplementary Table 3. List of primer sequences for real-time PCR and library construction.

Supplementary Table 4. Technical specifications of scCRISPR immune screens.

Supplementary Table 5: Statistical evaluation of k-NN scores for each cell category between the "Random" group and "Actual" group.

Supplementary Table 6. List of genes involved in response adoptive T cell transfer with the genetic perturbation

Supplementary Table 7. List of genes involved in response to anti-PD-1 with the genetic perturbation

Supplementary Table 8. List of significant changed genes involved in the interaction between the genetic perturbation and *in vitro* T cell treatment

Supplementary Table 9. List of significant changed genes involved in the interaction between the genetic perturbation and in *vivo* anti-PD-1 treatment
